# Assessing the performance of methods for copy number aberration detection from single-cell DNA sequencing data

**DOI:** 10.1371/journal.pcbi.1008012

**Published:** 2020-07-13

**Authors:** Xian F. Mallory, Mohammadamin Edrisi, Nicholas Navin, Luay Nakhleh

**Affiliations:** 1 Department of Computer Science, Rice University, Houston, Texas, United States of America; 2 Department of Computer Science, Florida State University, Tallahassee, Florida, United States of America; 3 Department of Genetics, the University of Texas M.D. Anderson Cancer Center, Houston, Texas, United States of America; University of Ottawa, CANADA

## Abstract

Single-cell DNA sequencing technologies are enabling the study of mutations and their evolutionary trajectories in cancer. Somatic copy number aberrations (CNAs) have been implicated in the development and progression of various types of cancer. A wide array of methods for CNA detection has been either developed specifically for or adapted to single-cell DNA sequencing data. Understanding the strengths and limitations that are unique to each of these methods is very important for obtaining accurate copy number profiles from single-cell DNA sequencing data. We benchmarked three widely used methods–Ginkgo, HMMcopy, and CopyNumber–on simulated as well as real datasets. To facilitate this, we developed a novel simulator of single-cell genome evolution in the presence of CNAs. Furthermore, to assess performance on empirical data where the ground truth is unknown, we introduce a phylogeny-based measure for identifying potentially erroneous inferences. While single-cell DNA sequencing is very promising for elucidating and understanding CNAs, our findings show that even the best existing method does not exceed 80% accuracy. New methods that significantly improve upon the accuracy of these three methods are needed. Furthermore, with the large datasets being generated, the methods must be computationally efficient.

## Author summary

Copy number aberrations, or CNAs, refer to evolutionary events that act on cancer genomes by deleting segments of the genomes or introducing new copies of existing segments. These events have been implicated in various types of cancer; consequently, their accurate detection could shed light on the initiation and progression of tumor, as well as on the development of potential targeted therapeutics. Single-cell DNA sequencing technologies are now producing the type of data that would allow such detection at the resolution of individual cells. However, to achieve this detection task, methods have to implement several steps of “data wrangling” and dealing with technical artifacts. In this work, we benchmarked three widely used methods for CNA detection from single-cell DNA data, namely Ginkgo, HMMcopy, and CopyNumber. To accomplish this study, we developed a novel simulator and devised a phylogeny-based measure of potentially erroneous CNA calls. We find that none of these methods has high accuracy, and all of them can be computationally very demanding. These findings call for the development of more accurate and more efficient methods for CNA detection from single-cell DNA data.

## Introduction

Acquired mutations are the main causes of cancer [1–3]. Copy number aberrations (CNAs) are one such type of acquired mutations and have been implicated in over-regulating oncogenes or down-regulating tumor suppressor genes [4]. Consequently, accurate detection of CNAs could hold a great promise to understanding some of the genetic underpinnings of cancer as well as developing targeted drugs. In the past two decades, a wide array of DNA technologies have been used to detect CNAs, among which the three most widely used are array Comparative Genomic Hybridization (aCGH), Next Generation Sequencing (NGS), and single-cell sequencing [5].

Array CGH [6] is a cytogenetic approach that uses fluorescent dyes on the test (tumor) and reference (normal) samples, and applies them to a microarray, which is an array of probes. Each probe is a DNA sequence that represents a region of interest. The size of a probe depends on the DNA sequence being used, and it varies from dozens of base pairs, such as oligonucleotides, to thousands of base pairs, such as bacterial artificial chromosomes. The probes from the paired samples, after being mixed together, hybridize at each target region. The fluorescence intensities can then be measured for both samples, and the ratio of the two is used to inform about CNAs of the test sample relative to the normal one. Array CGH data is advantageous in comprehensively detecting aneuploidies, amplifications, and deletions simultaneously. A few computational methods [7–9] have been developed to detect CNAs using aCGH data. DNAcopy [7] applies a modification of binary segmentation [10] called circular binary segmentation (CBS) to aCGH to overcome data noise, but it suffers from the problem of outliers [8, 9]. HMMcopy [8] was designed to ameliorate the problem of outliers and uses a Hidden Markov Model (HMM) to divide the genome into piecewise fixed segments in order to make inferences on CNAs. However, since aCGH data is limited in resolution and throughput [11], as well as suffers from a hybridization bias problem, it is not the optimal technology to detect CNAs for cancer samples.

Unlike aCGH, which obtains signal on only a limited number of genomic sites of interest, NGS technology makes it possible to survey the whole genome at a nucleotide-level resolution by sequencing millions of small fragments (reads) of the genome in parallel. By aligning the reads to an assembled reference genome, the reads that cover a position in the genome are counted to obtain the read depth at that position. Read depths at different regions of the genome can then be contrasted to assess hypothesized genomic locations where copy number gains and losses had occurred. NGS technologies suffer from high false positive rate compared with aCGH, due mainly to GC bias and the presence of repetitive regions [12, 13]. Even more challenging in the case of cancer genomes that are often aneuploid, contamination of normal cells may occur in the bulk tissue further complicating the task of estimating the absolute copy number from NGS data, i.e., the integer value representing the number of copies of a region on the genome. To overcome these challenges, a plethora of computational tools [12, 14–21] have been developed for detecting CNAs from NGS data. SeqCNA [12] filters out potentially false-positive CNAs and corrects GC content for a more accurate CNA detection. CNAseg [14] analyzes flowcell-to-flowcell variability to avoid false-positive CNAs. CNAnorm [15] addresses the normal contamination and aneuploidy of the tumor sample to infer CNAs accurately. Paired-end NGS data provides another modality in addition to the read depth to infer CNA, and a few bioinformatics tools use this, including, for example, CNVer [17], CNVnator [18], ReadDepth [19], and Mseq-CNV [20].

Although both aCGH and NGS data can be used to detect CNAs, they do not provide high-throughput data at the single-cell resolution that is ideal for understanding tumor growth. In particular, intratumor heterogeneity [22] is best understood by using data obtained from individual cells within the tumor tissue. Indeed, in the last ten years, the field has made great strides towards developing technologies for single-cell DNA sequencing. Data generated by these technologies can be analyzed to detect CNAs and other types of mutations in individual cells and individual clones within the heterogeneous tumor [23]. For example, DOP-PCR is a PCR amplification method that generates low-coverage data suitable for CNA detection in single-cell data [24–27]. However, it also suffers from uneven coverage and allelic dropout [23] that could lead to false-positive calls of CNAs. Beyond this method, three tools have been extensively applied to single-cell sequencing data for CNA detection: AneuFinder [28, 29], CopyNumber [30], and Ginkgo [31]. Like HMMcopy, AneuFinder uses a Hidden Markov Model (HMM) to determine the segmentation of the genome, i.e., to computationally segment the genome into non-overlapping regions so that each region has a homogeneous copy number, and the absolute copy number of each segment. CopyNumber [30] pools all the cells together for joint segmentation to improve boundary detection accuracy. Since cancer cells in the same subclone mostly share the same CNA boundaries, such a strategy can improve the nucleotide resolution of the boundary by implicitly amplifying the signal in the data. Ginkgo [31] uses Circular Binary Segmentation (CBS) [7] to segment the genome, followed by inferring the integer value of the absolute copy number. It is worth noting that some methods designed for aCGH and NGS data have also been extensively used on single-cell data, and this is especially true for HMMcopy [32–36]. As both AneuFinder and HMMcopy are HMM-based methods, we focus on HMMcopy as a representative of the HMM-based approach due to its wide application to single-cell sequencing data in multiple studies [32–36]. However, considering AneuFinder appears later than HMMcopy, we also benchmark its performance particularly on different ploidies (average copy number across the genome), as from our observation of HMMcopy, correctly inferring the ploidy level is a challenge to such HMM-based approaches. A more recent method is SCNV [37], which uses a bin-free segmentation method to do segmentation and copy number profiling. However, the method has not been widely applied to single-cell DNA studies. Most recently, Chisel [38] was introduced for detecting CNAs in single-cell sequencing data. With the help of a matched normal sample or identified normal single cells, Chisel phases CNAs by germline single nucleotide polymorphisms at a low coverage, and further infers allele- and haplotype-specific CNAs. Such allele- and haplotype-specific CNAs can subsequently help improve single-cell clustering and phylogenetic tree inference. As our study here does not deal with phasing, we did not include Chisel in our study. Among CopyNumber, Ginkgo, and HMMcopy, only CopyNumber utilized the pooled information from single-cell data. The other two methods can be equally well applied to bulk samples. Moreover, HMMcopy was designed for aCGH data originally, and thus does not take into account the specific error profiles that characterize single-cell sequencing data, such as low and uneven coverage, or the computational challenges that arise due to biological processes such as aneuploidy in a tumor single cell.

In this paper, we compared and benchmarked three methods that have been widely applied to CNA detection on single-cell DNA cancer data: Ginkgo, HMMcopy, and CopyNumber. We developed a simulator of cancer genome evolution in the presence of CNAs and used it to investigate the accuracy, running time and memory consumption of the three methods. We also investigated their performance on a real dataset and assessed their consistency. An important contribution of this paper is the use of phylogeny-based analysis of CNA calls to identify potentially erroneous ones. In particular, the use of a phylogeny helps identify regions that have witnessed a large number of mutations which could be further explored for potential error or some interesting biological explanation.

We found that in terms of the accuracy of the detected breakpoints (positions on the genome where segmentation occurs) and memory consumption, HMMcopy is the best of the three methods, and in terms of running time, it is slower than CopyNumber but faster than Ginkgo. However, when evaluated the methods in terms of the actual copy number profiles they detect, we found that Ginkgo is more accurate than HMMcopy; in fact, we found that HMMcopy is not stable at this task (paradoxically, CopyNumber does not detect actual copy numbers). In terms of accuracy, CopyNumber has a very poor performance. While Knouse *et al*. [34] assessed the performance of CBS and HMM-based methods on single-cell DNA sequencing data, their evaluation is limited to CNVs in brain and skin cells. Moreover, they did not investigate the effect of the ploidy on the accuracy of the methods. Our results highlight the need for developing new accurate and efficient methods for CNA detection from single-cell DNA data.

## Results

To better understand the strengths and limitations of current approaches for CNA detection from single-cell DNA sequencing data, we selected HMMcopy, Ginkgo, and CopyNumber using both simulation and realistic data.

For simulation, we designed three experiments to evaluate the performance of the three methods under different conditions. The first experiment was designed to evaluate the recall and precision of the CNA detection methods. We designed the simulation study in this experiment to produce single cells that have ideal read count variability and normal ploidy levels ranging between 2 and 3, so that we can learn how the methods perform when the data is relatively not challenging. The second experiment was designed to evaluate the performance of each method under a variety of ploidy levels. Specifically, we simulated single cells whose ploidies range from 1.5 to 5.26. We then compared the recall and precision of the three methods on the simulated data at different ploidies. The third experiment was designed to assess the performance of each method under different coverage variabilities. In particular, we simulated single cells whose coverage variabilities mimic those produced by three single-cell sequencing technologies (MALBAC, DOP-PCR and TnBC) [39] that have been used for CNA detection.

### Performance on simulated datasets

In the first experiment, we simulated the evolution of 10,000 cells from which we generated, through sampling without replacement, three 1000-cell datasets. For each cell, we simulated read data using a simulator that we developed (Methods). We then aligned the reads back to the reference genome using BWA with default parameters [40, 41]. Finally, we ran each of three methods on the resulting BAM files, and computed the recall and precision of each method based on the ground truth generated by the simulator.

**Fig 1:**
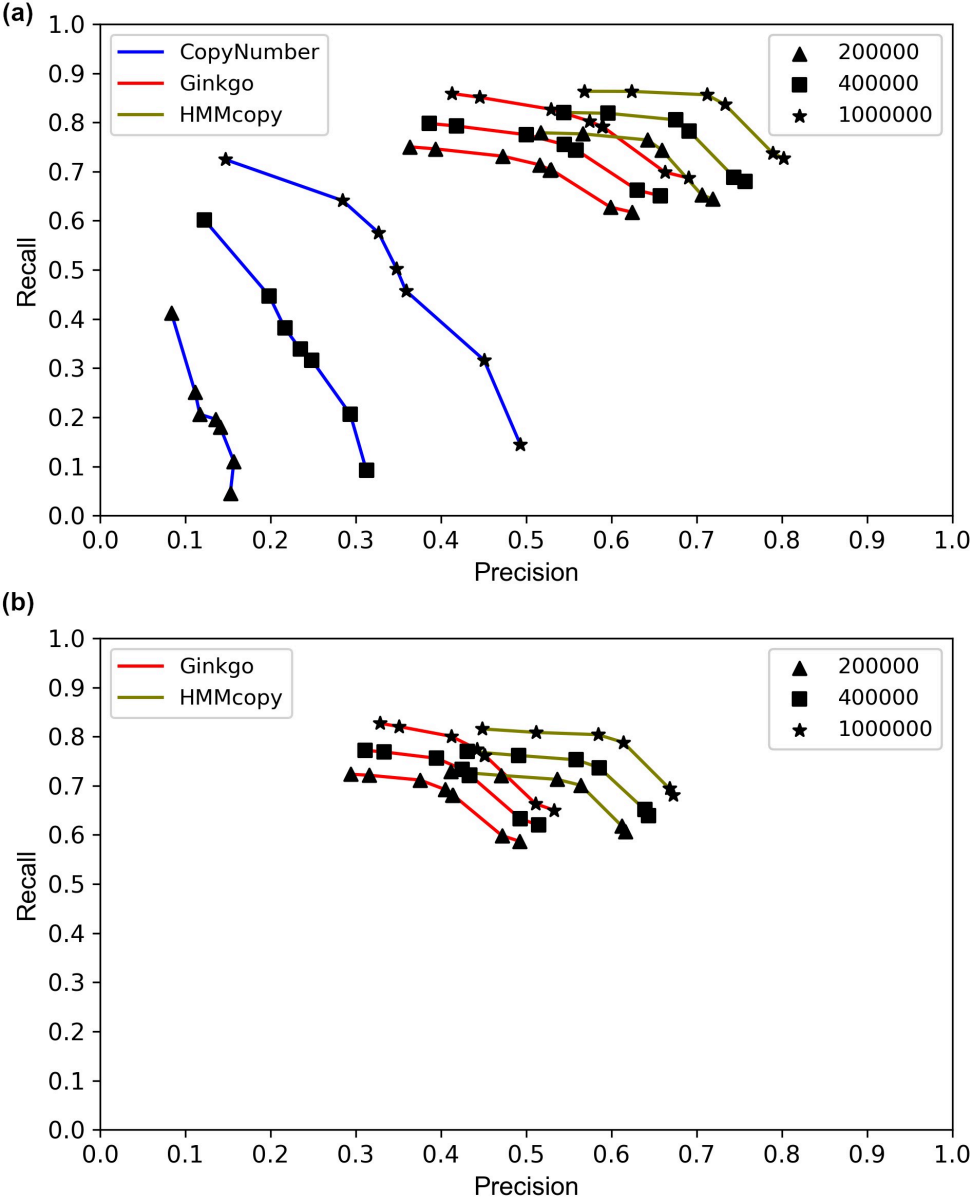
ROC curves of the three methods HMMcopy, Ginkgo, and CopyNumber. (a) Coarse-grained analysis results, and (b) fine-grained results. For each method, the results based on three thresholds of correctness are plotted. For HMMcopy, *nu*, which controls the suggested degree of freedom between states, was tuned to take on the values 0.01 (rightmost), 0.1, 2.1 (the tool’s default), 4, 10, and 20 (leftmost). For Ginkgo, *alpha*, which controls the significance level to accept a change point, was tuned to take on the values 1e-1000 (rightmost), 1e-100, 1e-10, 1e-5, 1e-4, 1e-3, 1e-2 (the tool’s default), 0.02 and 0.05 (leftmost). The dots corresponding to values 1e-5 and 1e-10 in coarse-grained analysis overlap. For CopyNumber, *gamma*, which is the weight of the penalty on changing a state, was tuned to take on the values 40 (rightmost, and the tool’s default), 10, 5, 4, 3, 2, and 1 (leftmost).

We assessed the methods’ performances in coarse- and fine-grained analyses. For the coarse-grained evaluation, we inspected the breakpoint positions as well as whether they were consistent with the ground truth in terms of the estimated gain/loss state (rather than the actual value) in the copy number. The predicted breakpoint is counted as consistent with the ground truth whenever it has the same status (i.e., the copy number increases or decreases) and its genomic location is within a certain distance of its counterpart in the ground truth. We varied the value of this distance to study the methods’ accuracies. Each ground truth breakpoint was matched by at most one predicted breakpoint to avoid double counting of the true-positive calls. For each method, we varied a parameter to obtain the receiver operator characteristic (ROC) curve, the details of which are described in the caption of Fig 1.

A preliminary analysis of CopyNumber on the data revealed that the method achieves extremely low recall and precision. Since CopyNumber pools all the cells together for breakpoint detection, we suspected that this poor performance owes mainly to the method’s lack of sensitivity in detecting breakpoints shared by only a small number of cells. Therefore, to allow for more meaningful comparison of CopyNumber to the other two methods, we eliminated breakpoint pairs shared by fewer than five cells in the ground truth and used the resulting new ground truth to evaluate CopyNumber’s recall and precision (but we did not filter the breakpoints for the other two methods). As Fig 1a shows, CopyNumber still has, by far, the poorest performance. We hypothesize that for a breakpoint to be detectable by CopyNumber, it needs to be shared by a large number of cells. We further checked this by calculating the number of cells sharing a breakpoint that is called or missed by CopyNumber, and found that there is a significant difference between the two sets (*p*-value < 9.019e-09 for Student’s t-test with mean 9.27 versus 5.35). We also observe that as the tolerance threshold for the detected breakpoint position increases, improvement in CopyNumber’s performance is much larger than the improvement in the performance of the other two methods. However, even with the most forgiving threshold (two breakpoints are considered to be the same if their positions are within 1 million basepairs of each other), CopyNumber still has poorer performance than the other two methods even under the most stringent threshold. Overall, the results in Fig 1a show that (1) HMMcopy generally outperforms the other two methods, with Ginkgo being a close second, and (2) that even HMMcopy’s best recall and precision are around 0.8 and 0.7, respectively.

In the fine-grained analysis, we focused on the agreement of the absolute copy numbers on both 5’ and 3’ of an inferred breakpoint with those of the ground truth, in addition to the requirements on gain/loss consistency and distance. Since CopyNumber does not predict the absolute copy numbers for an individual cell, it is not considered in this analysis. Surprisingly, HMMcopy’s prediction of the absolute copy number is not stable, leading to a bimodal distribution of both recall and precision (S1 Fig). We found that cells with low recall and precision correspond mainly to cases where HMMcopy made inaccurate estimates of the cells’ ploidies (S2 Fig). We then selected only those cells for which the ploidy was correctly predicted (i.e., 2 or 3), and plotted the ROC curve of HMMcopy on them. We found that HMMcopy performed generally better than Ginkgo (Fig 1b), which is in agreement with the coarse-grained analysis. The recall and precision for the two methods dropped, which is expected since the true positives and negatives are now measured most stringently. However, we observed that the difference in results between the coarse- and fine-grained analyses is not large, suggesting that once the breakpoint is found by these methods, predicting the absolute copy number can be attained quite accurately. This is especially true for Ginkgo whose ploidy prediction is stable.

Similar results were observed on the other two datasets (S3 and S4 Figs).

The results in Fig 1 were obtained under default parameters except for the parameters that were tuned to generate the ROC curves (*alpha*, *gamma*, and *nu*). However, we found that the value of parameter *strength* in HMMcopy has to be much larger than the default value in order to make the results more expected, i.e., increasing recall is accompanied with decreasing precision, and vice versa. We therefore set *strength* to be 10 million. According to HMMcopy’s users’ guide, *strength* is the parameter that controls how much the initial values of *e*, which controls the probability of extending a segment, remains the same throughout the iterations. We found that setting *strength* would help to have a good quality control of the result by making the initial setting of *e* last throughout all the iterations. Apart from parameters *nu* and *strength*, we found that *e* is also an important parameter in HMMcopy. The larger the value of *e*, the smaller the chance that the breakpoint is detected. To explore which combination can yield the best performance for HMMcopy, we varied both *e* and *nu* and calculated the F1 score. The performance of HMMcopy is the best when *nu* is 4 and *e* ≥ 0.999999 (S5 Fig).

**Fig 2:**
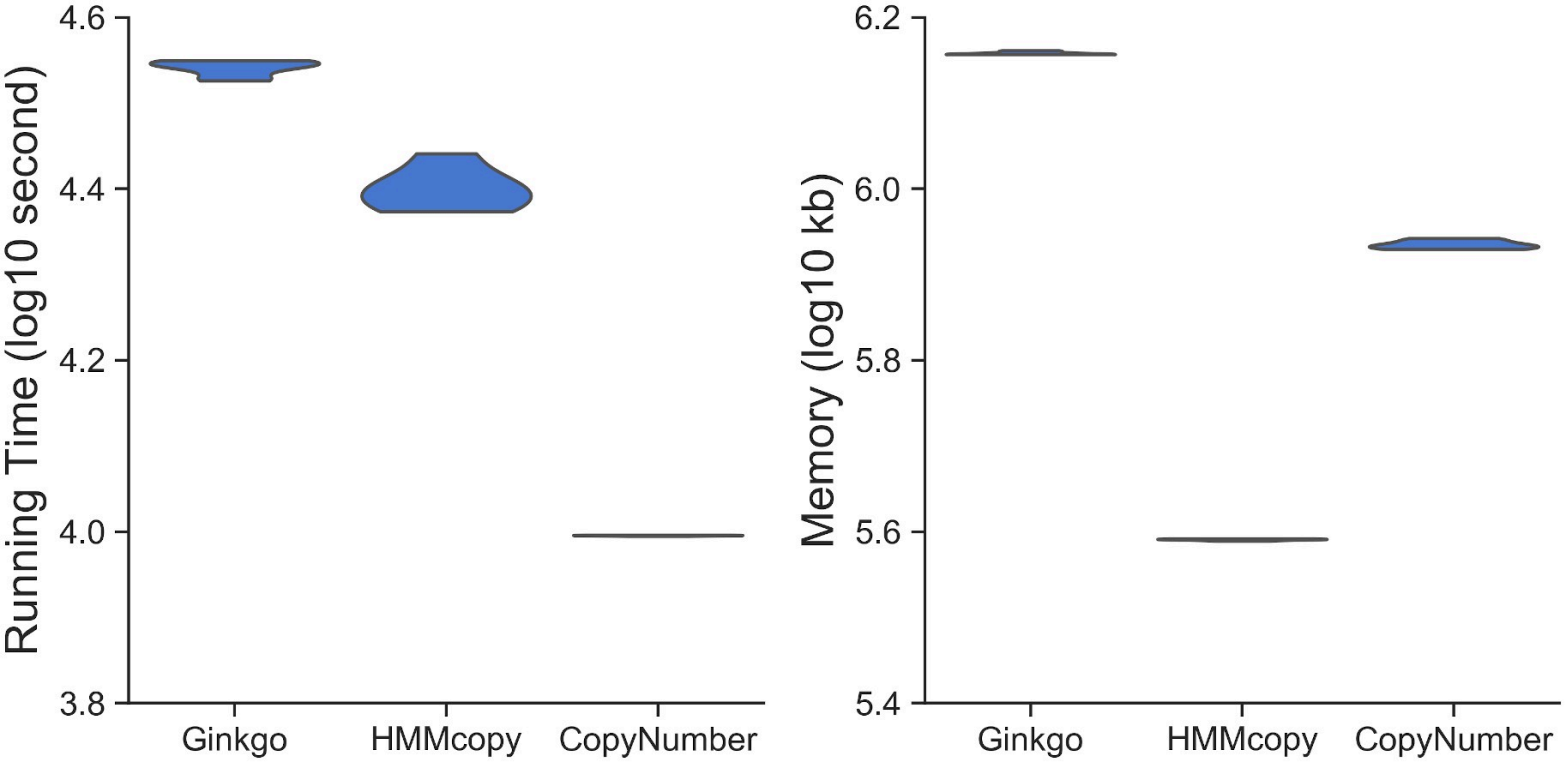
Computational requirements of Ginkgo, HMMcopy, and CopyNumber. Results are for analyzing a 1000-cell dataset on Intel(R) Xeon(R) CPU E5-2650 v2 whose clock speed is 2.60GHz. Left and right panels correspond to running time (in log10 of seconds) and memory consumption (in log10 of kb). The running time and memory were recorded for using different parameters as described in Fig 1. As Ginkgo’s running time increases more than twofold for *α* = 0.05, we treated it as an outlier and did not include this running time point in this plot.

We also analyzed the computational requirements in terms of running time and memory consumption for the three methods on a 1000-cell dataset (Fig 2). Ginkgo is the slowest among the three and CopyNumber is the fastest. HMMcopy is in between Ginkgo and CopyNumber in terms of running time. For memory consumption, Ginkgo is the most demanding of the three, followed by CopyNumber. HMMcopy is the lightest in terms of memory consumption. Note that in running Ginkgo, we eliminated the steps of generating figures such as heatmaps and copy number profile, so that these do not affect the running time and memory in comparison with the other two methods. For CopyNumber, an extra step is required to generate its input file. We used the intermediate result of HMMcopy, i.e., the GC corrected read count on each bin, as the input to CopyNumber. We take the time for calculating this intermediate file into account for CopyNumber for a fair comparison. Since CopyNumber processes all the cells together, we suspect that more cells will require more memory, whereas Ginkgo and HMMcopy’s memory requirements are not affected by the number of cells involved.

In summary, we found that HMMcopy is the most accurate in predicting breakpoints among the three. When HMMcopy’s prediction of ploidy is accurate, its recall and precision of predicting the absolute copy number are the best among the three methods. However, it is not as stable as Ginkgo in terms of the absolute copy number detection since its prediction of ploidy is wrong for many cells (49.4% for default values of *nu* and *e*). CopyNumber’s recall and precision are the worst of the three methods. Moreover, it cannot predict the absolute copy number for each individual cell, and thus is not as applicable as the other two methods.

### The effect of ploidy on performance

To test the robustness of the methods to different ploidies, we varied the ploidy by tuning the parameters that control whole chromosomal amplifications and the rate of deletion (see the “Methods” section). We varied the ploidy from 1.5 to 5. Specifically, we used 1.5, 2.1, 3, 3.8, and 5.26 (we refer to them as 1, 2, 3, 4, and 5, respectively, hereafter), and generated three datasets for each ploidy. We tuned the coverage parameter for each ploidy so that the total number of reads for different ploidies are approximately the same to avoid adding reads for larger genomes resulting from higher ploidies.

We ran each method using their default parameters (except the *strength* parameter in HMMcopy). Finding CopyNumber’s recall to be zero using the default *gamma*, we tuned *gamma* using the optimal value, i.e., 5, shown in Fig 1. We then found the recall greatly increased with this setting. Similar to the previous experiment, we again removed those breakpoint pairs shared by ≤ 5 cells from the ground truth for evaluating CopyNumber’s performance.

**Fig 3:**
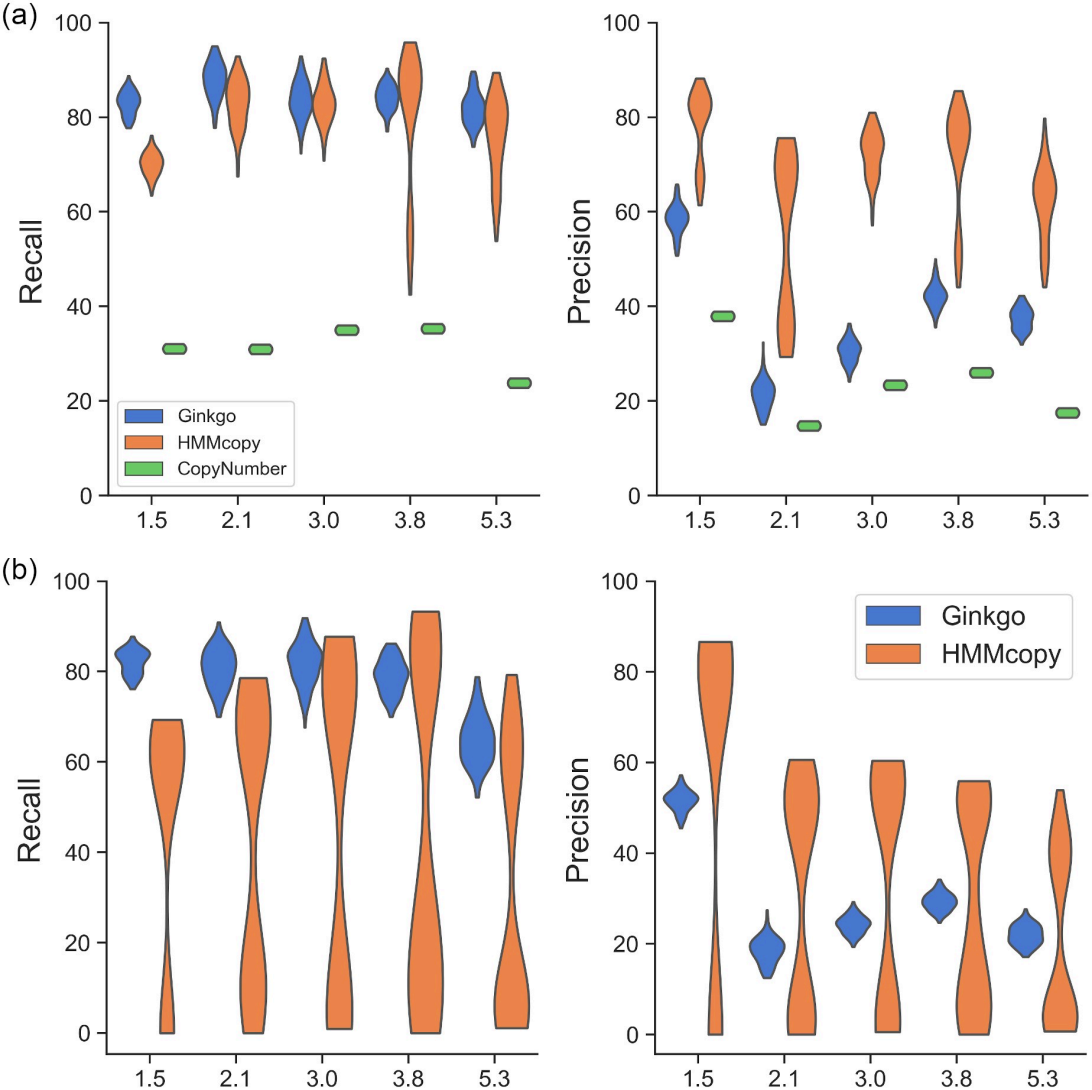
Recall and precision of Ginkgo, HMMcopy, and CopyNumber as functions of the ploidy. The ploidy level is varied and the results are based on the (a) coarse-grained and (b) fine-grained analyses. The ploidies of the simulated data were 1.5, 2.1, 3.0, 3.8, and 5.3.

We used different combinations of the parameters to simulate high-ploidy cells (details are in the “Methods” section), i.e., 4 and 5, and found that in the absence of odd and intermediate copy numbers, HMMcopy’s inference of the ploidy and absolute copy numbers were inaccurate (S6 Fig). This is also the case for Ginkgo in the absence of the odd copy numbers. However, despite the lack of intermediate copy numbers, Ginkgo correctly predicted absolute copy numbers for the case of ploidy = 5, showing that Ginkgo is more robust to changes in ploidy than HMMcopy in terms of predicting absolute copy numbers. In summary, the lack of odd or intermediate copy numbers in the data led to wrong predictions of absolute copy numbers. We then tuned the simulator’s parameters so that in higher ploidies there are odd and intermediate copy numbers to avoid the extremely hard cases for CNA detection (details are in the “Methods” section). Fig 3 shows the precision and recall for the three methods for coarse- and fine-grained analyses, respectively.

In the coarse-grained analysis, Ginkgo’s recall is > 0.8 in general, but its precision is relatively low (i.e., < 0.4) for ploidy 2 and 3. This is probably because it was affected by the variability of the read counts and over-segmented the genome. With similar recall, HMMcopy has higher precision at all ploidies. CopyNumber’s recall and precision are low (< 0.4) for all ploidies, with low recall and precision for ploidy 5 and low precision for ploidy 2.

In the fine-grained analysis, Ginkgo’s recall and precision dropped by about 10% as compared with the coarse-grained results. Its recall dropped the most for ploidy 5, indicating the challenge in accurately predicting the absolute copy number for high-ploidy cells. Although the odd and intermediate copy number states are present in this simulated data, HMMcopy’s precision and recall are still bimodally distributed for all ploidies.

As we observed cells whose incorrect ploidy prediction led to wrong prediction of absolute copy numbers in the previous experiment, these bimodal distributions further showed that such wrong prediction can widely occur to cells with different ploidies. Similar results were observed on the other two datasets (S7, S8, S9 and S10 Figs). We then plotted the predicted versus actual ploidies for all cells for five simulated ploidies on one dataset, and found that HMMcopy’s predicted ploidies deviate from the true ploidy for all simulated ploidies, whereas Ginkgo’s prediction of ploidy is relatively accurate (S11 and S12 Figs). Specifically, we noticed that when the ploidy is between 1.5 and 3, most of the incorrectly predicted ploidies accumulated at the values that are multiples of the actual ploidy. For example, when the actual ploidy is 2, the incorrectly predicted ploidies were at 4 and 6, as seen in the histogram in S12(c) Fig. When the actual ploidy is greater than 4, the incorrectly predicted ploidies centered around the actual ploidy but with a large deviation. While both phenomena are probably due to the wrong selection of the best-fitting ploidy, which can be further traced to a suboptimal scoring algorithm for selecting the best ploidies in HMMcopy, the first phenomenon of having multiples of the actual ploidy is possibly also due to that the algorithm mistook the noises as the intermediate copy numbers, leading to a much larger predicted ploidy. In addition, while both Ginkgo and HMMcopy adopt a post-segmentation step for inferring the ploidy, the fact that Ginkgo allows non-integer ploidy but HMMcopy does not may lead to their performance difference in inferring the ploidy.

Since AneuFinder and HMMcopy are both HMM-based methods but use different emission probability distributions (HMMcopy uses Gaussian whereas AneuFinder uses negative binomial), we also tested AneuFinder on datasets of varying ploidies (1.14.0, method=“edivisive”, binsizes = 2e5). We applied AneuFinder to the same dataset we described above and found that AneuFinder’s recall and precision are generally lower than that of HMMcopy’s, and the difference is extremely large when the ploidy is above 3. In particular, AneuFinder’s precision drops below 0.2 for ploidy at 4 and 5 with recall between 0.4 and 0.6 (S13 Fig) for coarse-grained analysis. In other words, at a lower recall, the precision of AneuFinder for ploidy above 3 is at least 50% smaller than those of HMMcopy’s. When the ploidy is between 2 and 3, while the recall of AneuFinder is close to that of HMMcopy, its precision is still much poorer than that of HMMcopy. For fine-grained analysis, AneuFinder’s recall further drops near 0.2 when the ploidy is 4, and 0.05 when the ploidy is 5. Its precision also drops near 0.05 when the ploidy is 5 (S14 Fig). In all, we conclude that although AneuFinder does not show bimodality on fine-grained analysis, its recall and precision are both much lower than those of HMMcopy especially at high-ploidy datasets.

### The effect of coverage on performance

**Fig 4:**
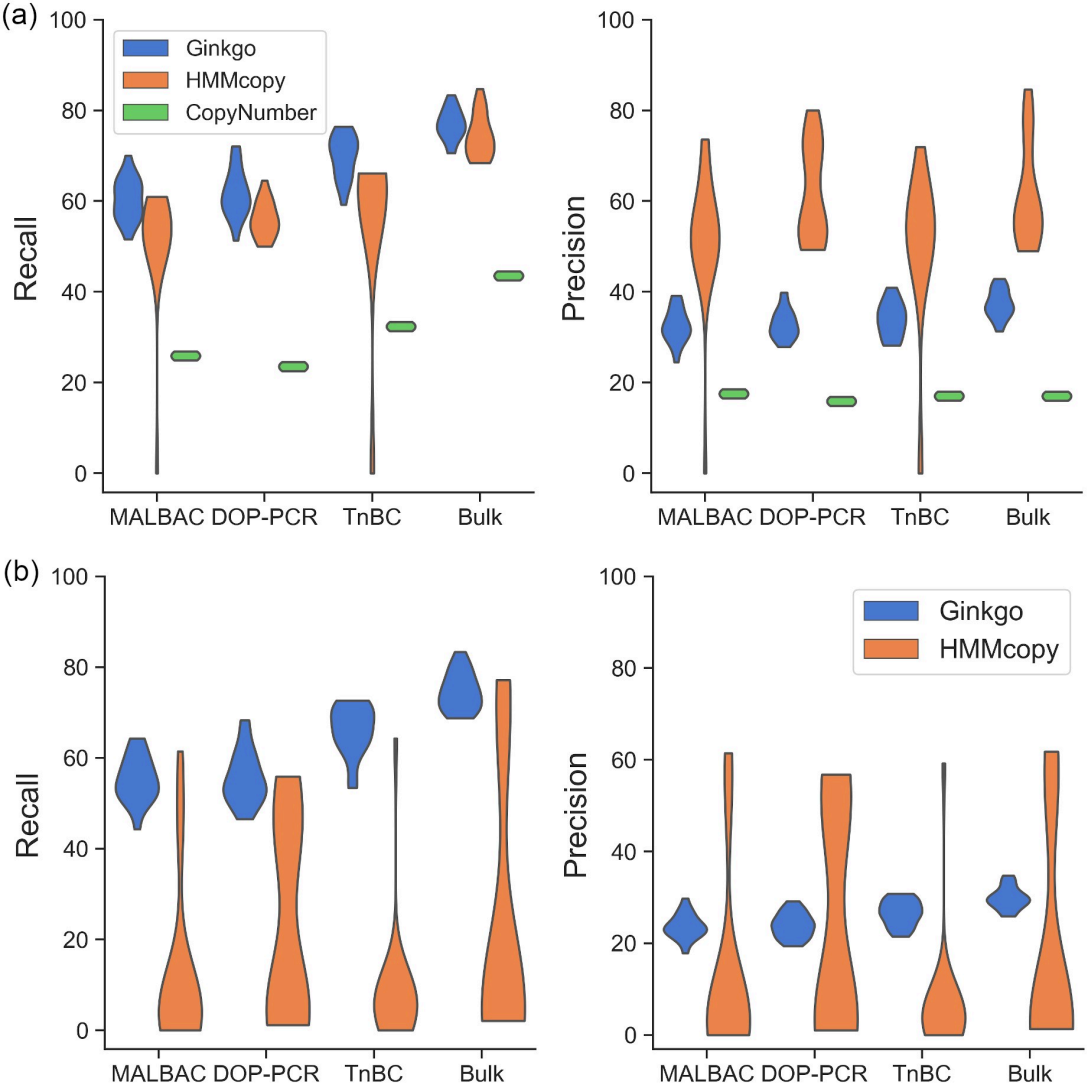
Recall and precision of Ginkgo, HMMcopy, and CopyNumber as functions of the coverage. The coverage is varied and the results are based on the (a) coarse-grained and (b) fine-grained analyses. The coverages are varied to mimic those produced by MALBAC, DOP-PCR, TnBC and Bulk sequencing.

To evaluate the performance of each method under different single-cell sequencing technologies, we sampled 20 cells from the simulated dataset and simulated their sequencing at four levels of coverage variabilities, corresponding to MALBAC, DOP-PCR, TnBC and Bulk sequencing (see details in the “Methods” section) and ran the three methods on each of them. We generated three datasets for each level of variability. Fig 4 shows the performance on one of the datasets. With decreasing variability, all three methods’ recall increased under both the coarse- and fine-grained analyses. Ginkgo’s and HMMcopy’s precisions increased with decreasing variability. CopyNumber’s precision, on the other hand, stays the same regardless of the coverage variability level, whereas its recall generally increases. In summary, better sequencing technology leads to better performance. The best that can be ever obtained (Bulk sequencing) is about 15% higher than the worst (MALBAC) for recall, and slightly higher for precision.

We looked into the copy number profiles in cases where HMMcopy’s precision and recall were effectively 0 (one such case is illustrated in S15 Fig). We found that choosing a wrong ploidy from the set of candidate ploidies by HMMcopy may result in a copy number profile in which all the segments are predicted to have the same absolute copy number, whereas the closest profile to the ground truth is among the reported non-optimal results. We observed that in such cases, the wrong choice of ploidy may affect both the segmentation and inference of the absolute copy number of those segments.

Similar results were observed on the other two repetitions (S16, S17, S18 and S19 Figs).

#### Performance on a real dataset

In real data analysis, due to the lack of ground truth, we evaluated the performance of the three methods in two ways. First, we evaluated the consistency among the three methods. The more overlap among the methods, the more consistent they are. Second, we inferred a maximum parsimony tree using PAUP [42] from the inferred copy number profiles and calculated the smallest number of copy number changes for each bin across all the branches of the tree, where the genome at the root of the tree is assumed to be diploid. The rationale for the latter way of assessing performance is that if the CNAs called by a method result (under a parsimony analysis) in a very large number of changes of the copy number at any bin, then one plausible explanation is error in the calls (another plausible prediction is that, for some reason, the locus corresponding to that bin has repeatedly gained and lost copies during the evolution of the cells which could be indicative of some interesting biological processes at play).

We downloaded single-cell DNA sequencing data of seven samples (the median number of cells in the seven samples is 47) from [43] (NCBI Sequence Read Archive under accession SRP114962) and selected those pre-treatment samples whose CNA profiles had not changed due to treatment compared with mid-treatment and post-treatment ones. We then ran the three methods with default parameters (except for the *strength* parameter in HMMcopy, as discussed above) on the single cells in each sample.

**Fig 5:**
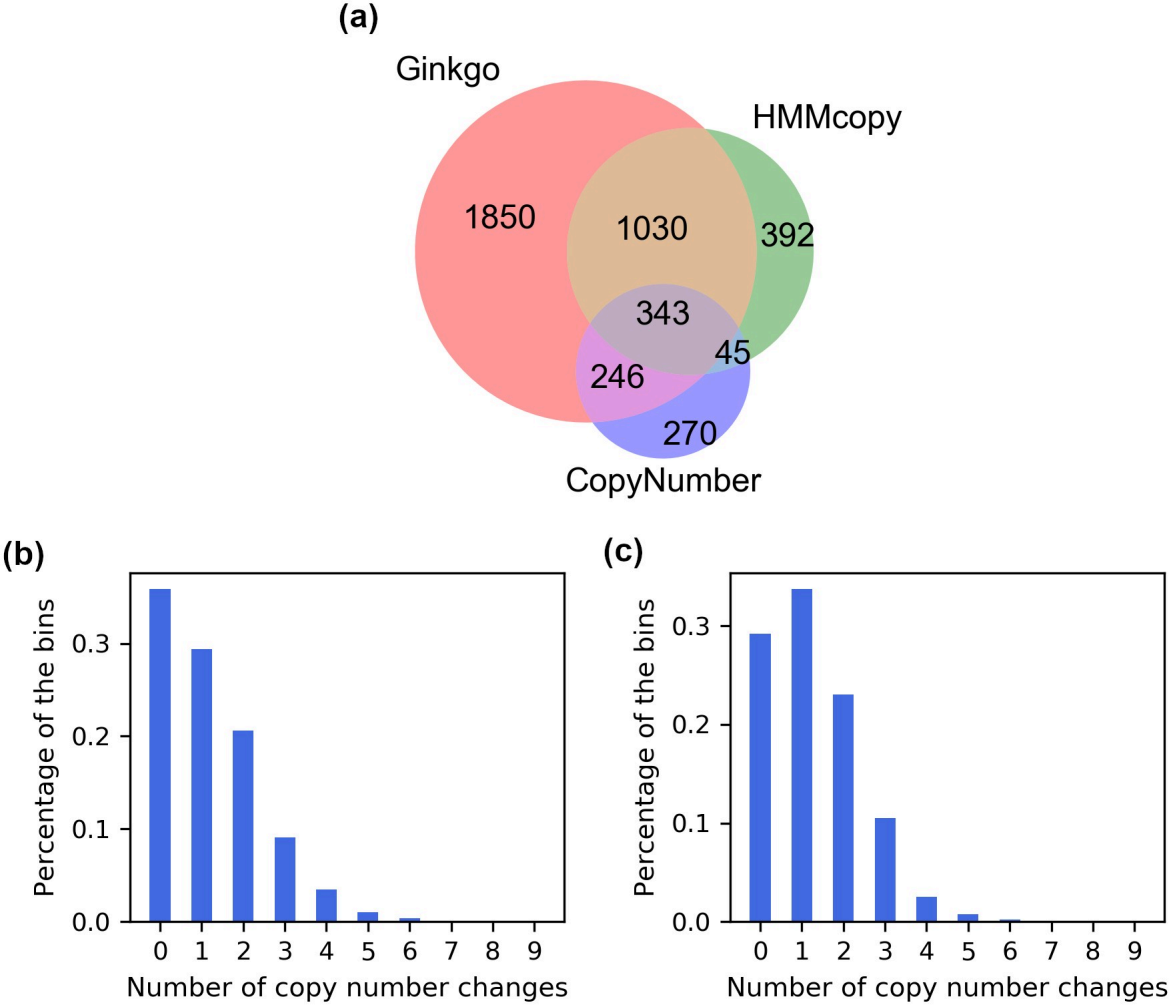
Comparison of HMMcopy, Ginkgo and CopyNumber on Sample 102 in [43]. (a) Venn diagram of the breakpoints from Ginkgo, HMMcopy and CopyNumber. Breakpoints from two methods are counted as overlapping if they are within 400,000bp of each other. (b) Distribution of the copy number changes (under a parsimony analysis) per bin based on the copy number profiles obtained by HMMcopy for the seven samples. (c) Distribution of the copy number changes (under a parsimony analysis) per bin based on the copy number profiles obtained by Ginkgo for the seven samples. For (b) and (c), a maximum parsimony tree was inferred from the copy number profiles of the cells, and the minimum number of copy number changes per bin along all the branches of the tree was computed by parsimony analysis. The percentages of the bins with each number of copy number changes are plotted.

For assessing accuracy, we generated for each sample a Venn diagram of the predictions based on all three methods, where predictions by two methods were deemed consistent according to the same rule we used in the simulation study (in assessing consistency between predictions and the ground truth). Fig 5a shows the results for Sample 102 (S20 Fig shows results for the other six samples). It can be seen that 47% of Ginkgo’s calls overlapped with the other two methods, leaving a large portion as unique calls. HMMcopy overlapped well with the other two methods, with 22% of unique calls. In particular, HMMcopy overlapped well with Ginkgo: 76% of HMMcopy’s calls overlapped with Ginkgo. CopyNumber’s overlap with Ginkgo was larger than that of HMMcopy (65% versus 43%). The overlap among the three methods is a very small portion of the union of all calls (8%), indicating a very large inconsistency among the three methods. From these results, we observe that HMMcopy performed the best among the three in breakpoint calling, if we consider consistency with other methods as a metric of quality, which is consistent with what we observed on the simulated data.

We then investigated the smallest number of changes required to explain the copy numbers detected by Ginkgo and HMMcopy (again, CopyNumber does not detect absolute copy numbers, which is why it is excluded in this analysis). To accomplish this, we inferred a phylogenetic tree from the inferred copy number profiles of the individual cells under the maximum parsimony criterion. We then inferred ancestral copy number profiles, also under the maximum parsimony criterion, and tabulated, for each bin, the number of changes that occurred along the branches of the tree. A bin that has a very large number of changes in the copy number could be pursued for further analysis. Two possible explanations of large number of changes are erroneous inferred copy numbers in this bin, or some real biological phenomenon that is worth pursuing further. Note that in this analysis we make the independent-bin assumption (copy number aberrations on each bin are independent of each other) and assume that bins undergo changes once at a time. More complex evolutionary models such as considering a group of neighboring bins together for a copy number aberration are beginning to emerge, but computing the parsimony score under those models is intractable [44]. Fig 5b and 5c show the distributions of copy number changes based on HMMcopy and Ginkgo’s results, respectively (S21 and S22 Figs show results for the other six samples). Interestingly, for Ginkgo, four out of seven samples (samples 102, 132, 152, and 302) showed a higher number of bins that have one copy number change than ones with no copy number changes. The other three samples (samples 126, 129, 615) have the most bins that had no copy number changes at all. Generally the number of bins that had copy number changes decreased with the increasing number of changes. On the other hand, based on the HMMcopy results, all samples showed much higher percentage of no copy number change than those with some copy number change.

## Discussion

Single-cell DNA sequencing data holds great promise for elucidating the complex biological processes that underly human diseases, including cancer. Copy number aberrations have been implicated in cancer, and their accurate detection from single-cell DNA sequencing data is of great potential to diagnosis and treatment of cancer. In this paper, we investigated the performance of three representative methods, Ginkgo, HMMcopy, and CopyNumber, that have been widely applied to single-cell sequencing cancer data for CNA detection.

We compared the three methods on simulated data generated under different settings that reflect varying degrees of complexity in the data. To accomplish this task, we developed a simulator that is flexible to simulate different scenarios and also mimic realistic data. We found that HMMcopy performs the best for breakpoint detection. However, HMMcopy is not stable in inferring the absolute copy number. Ginkgo performs well for both breakpoint detection and inference of the absolute copy number. CopyNumber is not as sensitive as the other two methods. We also looked into the performance of the three methods when ploidies were varied. We found that data with higher ploidies presented challenges for Ginkgo. HMMcopy is the most robust in terms of breakpoint detection among the three methods regardless of the ploidy, but its inference of the absolute copy number is not accurate for all ploidies. However, since the program yields copy number profiles for all possible integer ploidies, selecting the correct ploidy and obtaining the right copy number profiles will be trivial if given the pre-specified ploidy information, for example from DAPI staining. Both recall and precision of CopyNumber are the worst among the three methods. To explore the effect of technology artifacts on the accuracy of the methods, we simulated data that mimics the variability in coverage corresponding to MALBAC, DOP-PCR, TnBC, and Bulk. We found that all three methods’ recall generally increases with the improvement in the technology, with smaller observed change in their precision. We then applied the three methods to real data and evaluated their performance by analyzing the shared and unique detections they made as well as counting the total number of copy number changes that must be invoked based on their detections. We found a good amount of overlap in detections between Ginkgo and HMMcopy. We also found that HMMcopy’s detections result in fewer copy number changes than Ginkgo’s.

Our benchmarking study highlights several points. First, the ploidy and coverage of the genome under analysis affect the ability of a computational method to detect CNAs. Second, there is much more power in analyzing all cells in a data set simultaneously, as the fact that they all evolved from a common ancestral cell provides not only signal for the inference, but also accounts naturally for model complexity and regularizes the number of changes in copy numbers. Third, using algorithms and tools from the field of phylogenetics can help significantly in this area. As we demonstrated above on the biological data set, parsimony analysis of phylogenetic trees can be used to identify regions with large numbers of changes in the copy number, especially convergent changes, and those can be further inspected for determining whether the high rate of change reflects a biological process that is worth pursuing or it is due to sequencing or computational inference error artifacts. Finally, our results show that while existing methods are a good step in the right direction, there is need for developing more accurate methods for CNA detection, especially ones that are designed specifically to model the specifics of single-cell DNA sequencing.

## Methods

### Simulation

Two steps are involved in simulating reads for single-cell sequencing. First, the cell tree is generated, where the nodes are the cells, and the edges represent the parent-daughter cell relation. The leaf nodes represent the single cells that are sampled from the patient; the internal nodes represent the cells that existed in the past and were not sampled. We set the root node as a human normal genome without any CNA, assuming that all CNAs are somatic. The tree is simulated by the Beta-Splitting model (see below), which allows producing imbalanced trees, consistent with what was observed in the real data [45].

On each edge (except for those attached to the root of the tree; see below), we simulate a number of CNAs, the number of which corresponds to a Poisson distribution (by default, λ = 2). λ relates with the mutation rate which has been studied for two decades [46, 47]. There has not yet been a comprehensive knowledge of the mutation rate of CNAs, but according to the data from [43], we found there are about several dozens of CNAs in this dataset. The same can be found in a pan-cancer study [48]. Setting default λ to be 2 will lead to the similar number of CNAs at the leaves for a tree. The daughter cell of the edge inherits all CNAs in the parent node, in addition to its unique CNAs. To simulate a CNA, we randomly choose the allele, and the chromosome and position on the allele that CNA is going to occur. First, we sample the allele on which the CNA is going to occur from the paternal and maternal alleles according to a binomial distribution (default *p* = 0.5). We designed the simulator in a framework which keeps track of the allele at which the CNA occurs so that in our future work of simulating single nucleotide variations (SNV) simultaneously, the allele that is dropped due to the high allelic dropout rate can be traced. For the CNA size, we sample from an exponential distribution (default mean = 5Mbp), plus a minimum CNA size (default 2Mbp). We set the minimum CNA size by default to be 2Mbp because these CNAs are rare and commonly associated with disease [49], and also because of the limited resolution of single-cell data. The exponential distribution with mean 5Mbp is to render a wide range of CNA size. According to [34, 50], the larger the CNA size, the smaller the CNAs possibility. We choose copy number gain versus loss by a binomial distribution (default *p* = 0.5). We set the default parameter to be 0.5 so that copy number gain and loss are equally distributed. If a copy number gain is sampled, we sample from a geometric distribution (default *p* = 0.5) to determine the number of copies to be gained (mean = 1/*p*). This choice of a distribution is motivated by the observation that extremely high copy number gains are very rare and are often observed by double minutes amplification [51], which we do not take into account currently. Once a whole-genome DNA sequence is simulated with the CNAs, the gained copies are placed in tandem with the original copy. If a copy number loss is sampled, the whole sequence on that region of the allele is deleted.

The CNAs on the edges attached to the root node are simulated differently. In particular, clonal whole chromosomal amplifications can occur on these edge, as indicated in the punctuated evolution model observed in [45]. We simulate the chromosomal amplifications in addition to the focal CNAs. We set the probability of a chromosome to be amplified to be according to a binomial distribution (default *p* = 0.2). This default value is used so that while the whole chromosome amplification is introduced, 20% chromosomes in the genome will be changed. The number of the amplified copy is sampled from a geometric distribution (default mean is *p* = 1) multiplied by a value (default is 1) to amplify the copy numbers simulated without changing the distribution. The distribution of the whole chromosomal amplification can be turned off as an option.

At the edge to the root, we also add an option to allow more CNAs than the other edges. This is again to mimic a scenario of punctuated evolution [45]. To do that, we sample a value from a Poisson distribution (by default, λ = 4) which is the multiplier of the average number of the CNAs that occur to the edges other than the root. Thus the edge to the root has on average 4 times (default parameter) more focal CNAs than those of other edges. The higher this number, the more focal CNAs the edge to the root carries. This parameter is introduced to allow the user to simulate data that mimics the punctuated evolution model. However, due to the diversity of models that have been summarized for cancer evolution [52], users can turn off this option or tune the parameter λ so that the simulated data corresponds to their observation and experience. In our study, the value of λ was chosen according to the length of the trunk observed in [45].

Once we have the tree and the DNA sequences for all leaf nodes, we simulate the generation of read data from the genomes. Given the coverage of the genome (by default 0.04X), the simulator divides the genome into non-overlapping bins each of which has a default size of 200,000bp. To simulate the coverage variability observed in single-cell data, we use a Markov Chain Monte Carlo (MCMC) Metropolis-Hastings algorithm to determine a sequence of numbers of read pairs to be sampled for each bin.

An input of the variability information is a point on the Lorenz curve, whose X axis represents the percentage of the reads, and Y axis represents the percentage of the coverage. We transform it to a Beta distribution by Equations (1) and (2) in [53]. Through this transformation, we can sample read counts from a Beta distribution that corresponds to the given Lorenz curve. The followings are mathematical equations in [53] that are used to calculate the parameters (*α* and *β*) for the Beta distribution. In more detail, suppose *X* is a random variable whose cumulative distribution function *F* corresponds to a Beta distribution with parameters *α* and *β*. A point *x* sampled from this distribution has its corresponding X and Y positions on the Lorenz curve as *F*(*x*) and *ϕ*(*x*), where

$$\begin{array}{c} F\left(x\right)=I_{x}\left(\alpha ,\beta \right) \end{array}$$ (1)

and

$$\begin{array}{c} \phi \left(x\right)=I_{x}\left(\alpha +1,\beta \right) \end{array}$$ (2)

Given a point (*F*(*x*), *ϕ*(*x*)) on the Lorenz curve, we can calculate *α* and *β* for the Beta distribution.

Given the Beta distribution’s parameters, we can then sample read count for each bin by MCMC Metropolis-Hastings algorithm. Starting from the first bin whose read count is assigned as the expected coverage *x*_0_, we sample the next bin’s proposed read count *x*′ by a Gaussian distribution, and accept it if compared with the previous bin’s read count *x*_0_,

$$\begin{array}{c} \frac{I_{x^{\prime }}\left(\alpha ,\beta \right)\times \text{Gaus}\left(x^{\prime }|x_{0}\right)}{I_{x_{0}}\left(\alpha ,\beta \right)\times \text{Gaus}\left(x_{0}|x^{\prime }\right)}\leq u \end{array}$$ (3)

where **Gaus**(*x*′|*x*_0_) is the proposal probability of proposing *x*′ given *x*_0_, and *u* is the acceptance ratio. We set *u* to be 0.5 by default. We set the same standard deviations for **Gaus**(*x*′|*x*_0_) and **Gaus**(*x*_0_|*x*′), centered at *x*_0_ and *x*′, respectively. Thus the two Gaussian distributions canceled out. The rest term, $I_{x^{\prime }}/I_{x_{0}}\leq u$, controls how much the next bin’s read count differs from the current one. The read counts drawn are thus corresponding to a Beta distribution, and are simultaneously constrained by the acceptance ratio of the Metropolis-Hastings algorithm. This is to mimic the realistic data whose read coverage fluctuates, but the read count changes smoothly without sharp changes between neighboring bins.

### Running the programs

In all experiments, we eliminated reads that have mapping quality score < 40. We eliminated the cells that HMMcopy predicted as normal cells (predicted to be diploid and found no copy number aberration) in all experiments, the percentage of which was very small (< 0.2%).

### Parameters of simulator

The simulator is designed to be flexible, with user-specified parameters, as now describe.

#### Parameters for varying the ploidy level

To generate data with different ploidies, parameters associated with whole chromosomal amplification can be set for that purpose.

**-W (–whole-amp)** Controls whether there are whole chromosomal amplifications or not.
**-d (–del-rate)** The rate of copy number loss versus copy number gain.
**-C (–whole-amp-rate)** The possibility that a chromosome is selected to have whole chromosomal amplification.
**-E (–whole-amp-num)** For those chromosomes that are selected to be amplified, multiplying this number with the sampled value from a geometric distribution, whose *p* is “-J” described below, renders the final number of copies to be amplified.
**-J (–amp-num-geo-par)** The parameter *p* in the geometric distribution from which the number of copy of the chromosome to be amplified is sampled. Combination of **-J** and **-E** can make a variety of copy number distributions and make it convenient to attain higher copy number gains when necessary.

In our experiment above where we varied the ploidy level, we use a combination of these five parameters to generate data whose ploidies range from 1.5 to 5 as follows.

- **Ploidy = 1.5**: **-W 0 -d 1** No amplifications are allowed, and all copy number aberrations come from deletion.
- **Ploidy = 3**: **-W 1 -d 0.5 -C 0.5 -E 1 -J 1** Amplification is allowed, and the average number of amplification for the whole genome is 0.5 for one allele. The final ploidy is 3.
- **Ploidy = 4**, the case that lacks odd copy numbers: **-W 1 -d 0.5 -C 0.5 -E 2 -J 1** Amplification is allowed, and the average number of amplification for the whole genome is 1 for one allele. The final ploidy is 4. Note that since the parameter *p* in the geometric distribution (-J) is set to be one, the copy number is amplified by two for the allele that is selected for amplification. This causes the lack of intermediate copy numbers such as three, five, etc.
- **Ploidy = 3.8**, the case that has odd copy numbers: **-W 1 -d 0.5 -C 0.9 -E 1 -J 1** Amplification is allowed, and the average number of amplification for the whole genome is 0.9 for one allele. The final ploidy is 3.8. Compared with the previous case which lacks odd copy numbers, we increase the copy number by doubling 90% of the chromosomes. The following local copy number aberrations that are performed based on the amplified genome will then generate regions that have different copies, including the odd copies. In the absence of odd copy numbers, copy numbers 2, 4 and 6 will be considered as 1, 2, and 3 by any method. Thus, without copy numbers 1, 3 and 5, there is no way for a method to tell the correct absolute copy number.
- **Ploidy = 5**, the case that lacks intermediate copy numbers: **-W 1 -d 0.5 -C 0.5 -E 3 -J 1** Amplification is allowed, and the average number of amplifications for the whole genome is 1 for one allele. The final ploidy is 5. Note that since the parameter *p* in the geometric distribution (-J) is set to be one, the copy number is amplified by three for the allele that is selected for amplification. This causes a scenario where most of the copy numbers are two, five and eight.
- **Ploidy = 5.26**, the case that has intermediate copy numbers: **-W 1 -d 0.5 -C 0.9 -E 1 -J 0.55** Amplification is allowed, and the average number of amplification for the whole genome is 0.9 for one allele. Setting parameter *J* to be 0.55, the total amplified copy number for each allele is 1.63 (from 1/*p* × 0.9). The final ploidy is 5.26.

#### Parameters for varying the read count distribution

Since Lorenz curves have been used to evaluate the variability of read counts [32, 39, 54], we used the Lorenz curves reported in [39] for simulating variabilities at different levels. We sampled the read counts for each bin by the distribution (Beta distribution) corresponding to their Lorenz curves using a Markov Chain Metropolis-Hastings method (S23 Fig shows the Lorenz curves (left panel) and their corresponding Beta distributions (right panel) for the four technologies. The key parameters used for the Lorenz curves and Beta distributions corresponding to the four technologies are shown within the panels.).

### The Beta-splitting model

For generating the underlying evolutionary trees, we followed a generalization of the Blum-François Beta-splitting model [55] which is inspired by Aldous’ Beta-splitting model [56]. The construction of a tree based on this model [57] consists of two major steps: First, we generate two sequences of random values: *B* = (*b*_1_, *b*_2_, ⋯) and *U* = (*u*_1_, *u*_2_, ⋯), *B* is a sequence of independent and identically distributed (i.i.d.) random variables sampled from the B(α+1,β+1) distribution, and, *U* is a sequence of i.i.d. random variables with the uniform distribution on [0, 1]. We call $\left\{g_{i}={\left(u_{i},b_{i}\right)}_{i\in N}\right\}$ the *generating sequence* which is the basis of incremental construction of a tree. At the second step, we run the following algorithm on the random values generated at the first step. The process of constructing an evolutionary tree for *n* cells/leaves based on the Beta-splitting model with the parameters (*α*, *β*) combining these two steps is described in the following pseudocode.

**Algorithm 1** Algorithm for constructing a tree T with *n* leaves with the Beta parameters *α* and *β*.

1: **function** BuildBetaSplittingTree (*n*, *α*, *β*)

2:  Create the root of T

3:  T.root.label←(0,1)

4:  **for** *i* = 1⋯*n* **do**

5:   Sample $b_{i}∼B\left(\alpha +1,\beta +1\right)$

6:   Sample *u*_*i*_ ∼ *U*(0, 1)

7:   **for each** leaf∈T.leaves **do**

8:    (*x*, *y*) ← *leaf*.*label*

9:    **if** *u*_*i*_ ∈ [*x*, *y*] **then**

10:     *l* ← Create the left child of *leaf*

11:     *r* ← Create the right child of *leaf*

12:     *r*.*label* ← (*x* + (*y* − *x*)*b*_*i*_, *y*)

13:     *l*.*label* ← (*x*, *x* + (*y* − *x*)*b*_*i*_)

14:     *leaf*.*label* ← *i*

15:    **end if**

16:   **end for**

17:  **end for**

18:  **return** T

19: **end function**

### Software availability

The simulator has been implemented in Python and is freely available at https://github.com/compbiofan/SingleCellCNABenchmark.git, which also includes the scripts for regenerating the comparison results for both simulated and real datasets. The new version of HMMcopy was downloaded from https://github.com/shahcompbio/single_cell_pipeline/tree/master/single_cell/workflows/hmmcopy. The scripts to preprocess files for HMMcopy were downloaded from https://shahlab.ca/projects/hmmcopy_utils/. We use hg19 for all experiments in this manuscript and the mappability file used by HMMcopy was downloaded from http://genome.ucsc.edu/cgi-bin/hgFileUi?db=hg19&g=wgEncodeMapability. CopyNumber was downloaded from https://bioconductor.org/packages/release/bioc/html/copynumber.html. Ginkgo’s command line version which was used in this manuscript was downloaded from https://github.com/robertaboukhalil/ginkgo.

## Supporting information

S1 Fig Scatter plot of precision and recall of HMMcopy on the simulated large dataset. The color of the dots represent the ploidy that HMMcopy predicts. The upper and right histograms show the histogram of precision and recall, respectively, for all cells.
(PNG)

S2 Fig Precision and recall of HMMcopy on the simulated large dataset when ploidy estimation deviates from the ground truth which is 2.6. (a), (b) and (c) correspond to estimated ploidy 4, 5 and 6, respectively.
(PDF)

S3 Fig ROC curves of the three methods HMMcopy, Ginkgo, and CopyNumber on a second repetition. (a) Coarse-grained analysis results, and (b) fine-grained results for the second repetition. For each method, the results based on three thresholds of correctness are plotted. For HMMcopy, *nu*, which controls the suggested degree of freedom between states, was tuned to take on the values 0.01 (rightmost), 0.1, 2.1 (the tool’s default), 4, 10, and 20 (leftmost). For Ginkgo, *alpha*, which controls the significance level to accept a change point, was tuned to take on the values 1e-1000 (rightmost), 1e-100, 1e-10, 1e-5, 1e-4, 1e-3, 1e-2 (the tool’s default), 0.02 and 0.05 (leftmost). The dots corresponding to values 1e-5 and 1e-10 in coarse-grained analysis overlap. For CopyNumber, *gamma*, which is the weight of the penalty on changing a state, was tuned to take on the values 40 (rightmost, and the tool’s default), 10, 5, 4, 3, 2, and 1 (leftmost).
(PNG)

S4 Fig ROC curves of the three methods HMMcopy, Ginkgo, and CopyNumber on a third repetition. (a) Coarse-grained analysis results, and (b) fine-grained results for the third repetition. For each method, the results based on three thresholds of correctness are plotted. For HMMcopy, *nu*, which controls the suggested degree of freedom between states, was tuned to take on the values 0.01 (rightmost), 0.1, 2.1 (the tool’s default), 4, 10, and 20 (leftmost). For Ginkgo, *alpha*, which controls the significance level to accept a change point, was tuned to take on the values 1e-1000 (rightmost), 1e-100, 1e-10, 1e-5, 1e-4, 1e-3, 1e-2 (the tool’s default), 0.02 and 0.05 (leftmost). The dots corresponding to values 1e-5 and 1e-10 in coarse-grained analysis overlap. For CopyNumber, *gamma*, which is the weight of the penalty on changing a state, was tuned to take on the values 40 (rightmost, and the tool’s default), 10, 5, 4, 3, 2, and 1 (leftmost).
(PNG)

S5 Fig Plot of F1 scores of HMMcopy based on varying *nu* and *e* on one simulated dataset (1000 cells). On *nu*, 0, 1, 2, 3, 4, 5 and 6 represent 0.01, 0.1, 1, 2.1 (default), 4, 10 and 20. On *e*, 0, 1, 2, 3, and 4 represent 0.99, 0.9999, 0.999999, 0.99999999 (default), and 0.9999999999. It can be seen when *nu* is 4, and *e* is ≥ 0.999999, F1 score reaches the maximum.
(PNG)

S6 Fig Example of two cells that lack odd copy numbers (top panels, simulated ploidy = 4) and intermediate copy numbers (bottom panels, simulated ploidy = 5), respectively. The left and right panels show HMMcopy and Ginkgo’s results, respectively. For each panel, X and primary Y axes represent the whole genome segmented into bins and the raw read count for each bin, respectively. The secondary Y axis represents the absolute copy number. Black dots represent the raw read count for each bin. Red and blue horizontal lines represent the ground truth of the absolute copy number and the inferred ones from HMMcopy and Ginkgo. Due to the lack of odd copy numbers, both HMMcopy and Ginkgo incorrectly predicted absolute copy number for the case of ploidy = 4. Due to the lack of intermediate copy number, HMMcopy incorreclty predicted absolute copy number for the case of ploidy = 5. Despite the lack of intermediate copy numbers, Ginkgo correctly predicted absolute copy number for the case of ploidy = 5 (red and blue lines overlap in this case).
(PNG)

S7 Fig Recall and precision of Ginkgo, HMMcopy, and CopyNumber on varying ploidy level based on the coarse-grained analysis on a second repetition. The ploidies of the simulated data were 1.5, 2.1, 3.0, 3.8, and 5.3.
(PNG)

S8 Fig Recall and precision of Ginkgo and HMMcopy on varying ploidy level based on the fine-grained analysis on a second repetition. The ploidies of the simulated data were 1.5, 2.1, 3.0, 3.8, and 5.3.
(PNG)

S9 Fig Recall and precision of Ginkgo, HMMcopy, and CopyNumber on varying ploidy level based on the coarse-grained analysis on a third repetition. The ploidies of the simulated data were 1.5, 2.1, 3.0, 3.8, and 5.3.
(PNG)

S10 Fig Recall and precision of Ginkgo and HMMcopy on varying ploidy level based on the fine-grained analysis on a third repetition. The ploidies of the simulated data were 1.5, 2.1, 3.0, 3.8, and 5.3.
(PNG)

S11 Fig Comparison of Ginkgo’s predicted and actual ploidy on the varying ploidies. (a) A summary of all ploidies. Dots in purple, green, orange, red and blue represent the simulated ploidies at 1.5, 2.1, 3.0, 3.8 and 5.3. (b)-(f) Scatter plot of the predicted and actual ploidies for the five varying ploidies mentioned in (a) with their corresponding colors. Ploidy was calculated as the average copy number of the whole genome. On the right of each subplot, a histogram of the predicted ploidies is drawn to show the percentage of each predicted value. For (a)-(f), X and Y axis are the actual and predicted ploidies, respectively.
(PNG)

S12 Fig Comparison of HMMcopy’s predicted and actual ploidy on the varying ploidies. (a) A summary of all ploidies. Dots in purple, green, orange, red and blue represent the simulated ploidies at 1.5, 2.1, 3.0, 3.8 and 5.3. (b)-(f) Scatter plot of the predicted and actual ploidies for the five varying ploidies mentioned in (a) with their corresponding colors. Ploidy was calculated as the average copy number of the whole genome. On the right of each subplot, a histogram of the predicted ploidies is drawn to show the percentage of each predicted value. For (a)-(f), X and Y axis are the actual and predicted ploidies, respectively.
(PNG)

S13 Fig Recall and precision of AneuFinder on varying ploidy on coarse-grained analysis. The ploidies of the simulated data were 1.5, 2.1, 3.0, 3.8, and 5.3.
(PNG)

S14 Fig Recall and precision of AneuFinder on varying ploidy on fine-grained analysis. The ploidies of the simulated data were 1.5, 2.1, 3.0, 3.8, and 5.3.
(PNG)

S15 Fig An example of when wrong selection of ploidy in HMMcopy leads to wrong estimation of breakpoints. X and primary Y axes represent the whole genome segmented into bins and the raw read count for each bin, respectively. The secondary Y axis represents the absolute copy number. Black dots represent the raw read count for each bin. Red and blue horizontal lines represent the copy number profiles of HMMcopy on the condition when its ploidy estimation is 2 and 1, respectively, the latter of which was selected as the optimal ploidy by HMMcopy.
(PDF)

S16 Fig Recall and precision of Ginkgo, HMMcopy, and CopyNumber on varying coverages based on the coarse-grained analysis on a second repetition. The coverages are varied to mimic those produced by MALBAC, DOP-PCR, TnBC and Bulk sequencing.
(PNG)

S17 Fig Recall and precision of Ginkgo and HMMcopy on varying coverages based on the fine-grained analysis on a second repetition. The coverages are varied to mimic those produced by MALBAC, DOP-PCR, TnBC and Bulk sequencing.
(PNG)

S18 Fig Recall and precision of Ginkgo, HMMcopy, and CopyNumber on varying coverages based on the coarse-grained analysis on a third repetition. The coverages are varied to mimic those produced by MALBAC, DOP-PCR, TnBC and Bulk sequencing.
(PNG)

S19 Fig Recall and precision of Ginkgo and HMMcopy on varying coverages based on the fine-grained analysis on a third repetition. The coverages are varied to mimic those produced by MALBAC, DOP-PCR, TnBC and Bulk sequencing.
(PNG)

S20 Fig Venn diagram of the breakpoints from Ginkgo, HMMcopy and CopyNumber for the rest of the six samples. Breakpoints from two methods are counted as overlapped ones if they are within 400,000bp.
(PNG)

S21 Fig Distribution of the copy number changes (under a parsimony analysis) per bin based on the copy number profiles obtained by HMMcopy for the seven samples. A maximum parsimony tree was inferred from the copy number profiles of the cells, and the minimum number of copy number changes per bin along all the branches of the tree was computed by parsimony analysis. The percentages of bins with each number of copy number changes are plotted.
(PNG)

S22 Fig Distribution of the copy number changes (under a parsimony analysis) per bin based on the copy number profiles obtained by Ginkgo for the seven samples. A maximum parsimony tree was inferred from the copy number profiles of the cells, and the minimum number of copy number changes per bin along all the branches of the tree was computed by parsimony analysis. The percentages of bins with each number of copy number changes are plotted.
(PNG)

S23 Fig Left and right panels show the Lorenz curves and Beta distributions, respectively. The four rows represent four sequencing technologies: MALBAC, DOP-BAC, TnBC and Bulk. The parameters of generating Lorenz curve were learned from [39]. The parameters of generating Beta distributions were learned from Eqs 1 and 2 in the main text.
(PDF)

## References

[1] Feuk L, Carson AR, Scherer SW. Structural variation in the human genome. Nature Reviews Genetics. 2006;7(2):85 16418744 10.1038/nrg1767

[2] Sharp AJ, Cheng Z, Eichler EE. Structural variation of the human genome. Annu Rev Genomics Hum Genet. 2006;7:407–442. 16780417 10.1146/annurev.genom.7.080505.115618

[3] Lupski JR, et al Structural variation in the human genome. New England Journal of Medicine. 2007;356(11):1169 17360997 10.1056/NEJMcibr067658

[4] Beroukhim R, Mermel CH, Porter D, Wei G, Raychaudhuri S, Donovan J, et al The landscape of somatic copy-number alteration across human cancers. Nature. 2010;463(7283):899 10.1038/nature08822 20164920 PMC2826709

[5] Li W, Olivier M. Current analysis platforms and methods for detecting copy number variation. Physiological genomics. 2012;45(1):1–16. 10.1152/physiolgenomics.00082.2012 23132758 PMC3544484

[6] Carter NP. Methods and strategies for analyzing copy number variation using DNA microarrays. Nature genetics. 2007;39(7s):S16 10.1038/ng2028 17597776 PMC2697494

[7] Olshen AB, Venkatraman E, Lucito R, Wigler M. Circular binary segmentation for the analysis of array-based DNA copy number data. Biostatistics. 2004;5(4):557–572. 15475419 10.1093/biostatistics/kxh008

[8] Shah SP, Xuan X, DeLeeuw RJ, Khojasteh M, Lam WL, Ng R, et al Integrating copy number polymorphisms into array CGH analysis using a robust HMM. Bioinformatics. 2006;22(14):e431–e439. 16873504 10.1093/bioinformatics/btl238

[9] Ha G, Roth A, Lai D, Bashashati A, Ding J, Goya R, et al Integrative analysis of genome-wide loss of heterozygosity and monoallelic expression at nucleotide resolution reveals disrupted pathways in triple-negative breast cancer. Genome research. 2012;22(10):1995–2007. 10.1101/gr.137570.112 22637570 PMC3460194

[10] Sen A, Srivastava MS. On tests for detecting change in mean. The Annals of Statistics. 1975;3(1):98–108.

[11] Czyż ZT, Hoffmann M, Schlimok G, Polzer B, Klein CA. Reliable single cell array CGH for clinical samples. PloS one. 2014;9(1):e85907 10.1371/journal.pone.0085907 24465780 PMC3897541

[12] Mosen-Ansorena D, Telleria N, Veganzones S, De la Orden V, Maestro ML, Aransay AM. seqCNA: an R package for DNA copy number analysis in cancer using high-throughput sequencing. BMC genomics. 2014;15(1):178 10.1186/1471-2164-15-178 24597965 PMC4022175

[13] Jang H, Lee H. Multiresolution correction of GC bias and application to identification of copy number alterations. Bioinformatics. 2019. 10.1093/bioinformatics/btz174 30865265

[14] Ivakhno S, Royce T, Cox AJ, Evers DJ, Cheetham RK, Tavaré S. CNAseg—a novel framework for identification of copy number changes in cancer from second-generation sequencing data. Bioinformatics. 2010;26(24):3051–3058. 20966003 10.1093/bioinformatics/btq587

[15] Gusnanto A, Wood HM, Pawitan Y, Rabbitts P, Berri S. Correcting for cancer genome size and tumour cell content enables better estimation of copy number alterations from next-generation sequence data. Bioinformatics. 2011;28(1):40–47. 22039209 10.1093/bioinformatics/btr593

[16] Boeva V, Popova T, Bleakley K, Chiche P, Cappo J, Schleiermacher G, et al Control-FREEC: a tool for assessing copy number and allelic content using next-generation sequencing data. Bioinformatics. 2011;28(3):423–425. 10.1093/bioinformatics/btr670 22155870 PMC3268243

[17] Medvedev P, Fiume M, Dzamba M, Smith T, Brudno M. Detecting copy number variation with mated short reads. Genome research. 2010;20(11):1613–1622. 10.1101/gr.106344.110 20805290 PMC2963824

[18] Abyzov A, Urban AE, Snyder M, Gerstein M. CNVnator: an approach to discover, genotype, and characterize typical and atypical CNVs from family and population genome sequencing. Genome research. 2011;21(6):974–984. 10.1101/gr.114876.110 21324876 PMC3106330

[19] Miller CA, Hampton O, Coarfa C, Milosavljevic A. ReadDepth: a parallel R package for detecting copy number alterations from short sequencing reads. PloS one. 2011;6(1):e16327 10.1371/journal.pone.0016327 21305028 PMC3031566

[20] Malekpour SA, Pezeshk H, Sadeghi M. MSeq-CNV: accurate detection of Copy Number Variation from Sequencing of Multiple samples. Scientific reports. 2018;8(1):4009 10.1038/s41598-018-22323-8 29507384 PMC5838159

[21] Chiang DY, Getz G, Jaffe DB, O’kelly MJ, Zhao X, Carter SL, et al High-resolution mapping of copy-number alterations with massively parallel sequencing. Nature methods. 2009;6(1):99 10.1038/nmeth.1276 19043412 PMC2630795

[22] Nowell PC. The clonal evolution of tumor cell populations. Science. 1976;194(4260):23–28. 959840 10.1126/science.959840

[23] Navin NE. Cancer genomics: one cell at a time. Genome biology. 2014;15(8):452 10.1186/s13059-014-0452-9 25222669 PMC4281948

[24] Navin N, Kendall J, Troge J, Andrews P, Rodgers L, McIndoo J, et al Tumour evolution inferred by single-cell sequencing. Nature. 2011;472(7341):90 10.1038/nature09807 21399628 PMC4504184

[25] Carter NP, Bebb CE, Nordenskjo M, Ponder BA, Tunnacliffe A, et al Degenerate oligonucleotide-primed PCR: general amplification of target DNA by a single degenerate primer. Genomics. 1992;13(3):718–725. 1639399 10.1016/0888-7543(92)90147-k

[26] Arneson N, Hughes S, Houlston R, Done S. Whole-genome amplification by degenerate oligonucleotide primed PCR (DOP-PCR). Cold Spring Harbor Protocols. 2008;2008(1):pdb–prot4919. 10.1101/pdb.prot4919 21356673

[27] Baslan T, Kendall J, Rodgers L, Cox H, Riggs M, Stepansky A, et al Genome-wide copy number analysis of single cells. Nature protocols. 2012;7(6):1024 10.1038/nprot.2012.039 22555242 PMC5069701

[28] Bakker B, Taudt A, Belderbos ME, Porubsky D, Spierings DC, de Jong TV, et al Single-cell sequencing reveals karyotype heterogeneity in murine and human malignancies. Genome biology. 2016;17(1):115 10.1186/s13059-016-0971-7 27246460 PMC4888588

[29] van den Bos H, Spierings DC, Taudt A, Bakker B, Porubskỳ D, Falconer E, et al Single-cell whole genome sequencing reveals no evidence for common aneuploidy in normal and Alzheimer’s disease neurons. Genome biology. 2016;17(1):116 10.1186/s13059-016-0976-2 27246599 PMC4888403

[30] Nilsen G, Liestøl K, Van Loo P, Vollan HKM, Eide MB, Rueda OM, et al Copynumber: efficient algorithms for single-and multi-track copy number segmentation. BMC genomics. 2012;13(1):591 10.1186/1471-2164-13-591 23442169 PMC3582591

[31] Garvin T, Aboukhalil R, Kendall J, Baslan T, Atwal GS, Hicks J, et al Interactive analysis and assessment of single-cell copy-number variations. Nature methods. 2015;12(11):1058 10.1038/nmeth.3578 26344043 PMC4775251

[32] Laks E, Zahn H, Lai D, McPherson A, Steif A, Brimhall J, et al Resource: Scalable whole genome sequencing of 40,000 single cells identifies stochastic aneuploidies, genome replication states and clonal repertoires. bioRxiv. 2018; p. 411058.

[33] Zahn H, Steif A, Laks E, Eirew P, VanInsberghe M, Shah SP, et al Scalable whole-genome single-cell library preparation without preamplification. Nature methods. 2017;14(2):167 28068316 10.1038/nmeth.4140

[34] Knouse KA, Wu J, Amon A. Assessment of megabase-scale somatic copy number variation using single-cell sequencing. Genome research. 2016;26(3):376–384. 10.1101/gr.198937.115 26772196 PMC4772019

[35] Harmanci AS, Harmanci AO, Zhou X. CaSpER identifies and visualizes CNV events by integrative analysis of single-cell or bulk RNA-sequencing data. Nature Communications. 2020;11(1):1–16. 10.1038/s41467-019-13779-x PMC6941987 31900397

[36] Wang Y, Guo L, Feng L, Zhang W, Xiao T, Di X, et al Single nucleotide variant profiles of viable single circulating tumour cells reveal CTC behaviours in breast cancer. Oncology reports. 2018;39(5):2147–2159. 10.3892/or.2018.6325 29565466 PMC5928770

[37] Wang X, Chen H, Zhang NR. DNA copy number profiling using single-cell sequencing. Briefings in bioinformatics. 2017;19(5):731–736. 10.1093/bib/bbx004 PMC6171490 28159966

[38] Zaccaria S, Raphael BJ. Characterizing the allele-and haplotype-specific copy number landscape of cancer genomes at single-cell resolution with CHISEL. bioRxiv. 2019; p. 837195.

[39] Xi L, Belyaev A, Spurgeon S, Wang X, Gong H, Aboukhalil R, et al New library construction method for single-cell genomes. PloS one. 2017;12(7):e0181163 10.1371/journal.pone.0181163 28723968 PMC5517011

[40] Li H, Durbin R. Fast and accurate short read alignment with Burrows–Wheeler transform. bioinformatics. 2009;25(14):1754–1760. 10.1093/bioinformatics/btp324 19451168 PMC2705234

[41] Li H. Aligning sequence reads, clone sequences and assembly contigs with BWA-MEM. arXiv preprint arXiv:13033997. 2013.

[42] Swofford DL. Paup*: Phylogenetic analysis using parsimony (and other methods) 4.0. B5. 2001.

[43] Kim C, Gao R, Sei E, Brandt R, Hartman J, Hatschek T, et al Chemoresistance evolution in triple-negative breast cancer delineated by single-cell sequencing. Cell. 2018;173(4):879–893. 10.1016/j.cell.2018.03.041 29681456 PMC6132060

[44] El-Kebir M, Raphael BJ, Shamir R, Sharan R, Zaccaria S, Zehavi M, et al Complexity and algorithms for copy-number evolution problems. Algorithms for Molecular Biology. 2017;12(1):13 10.1186/s13015-017-0103-2 28515774 PMC5433102

[45] Gao R, Davis A, McDonald TO, Sei E, Shi X, Wang Y, et al Punctuated copy number evolution and clonal stasis in triple-negative breast cancer. Nature genetics. 2016;48(10):1119 10.1038/ng.3641 27526321 PMC5042845

[46] Martincorena I, Raine KM, Gerstung M, Dawson KJ, Haase K, Van Loo P, et al Universal patterns of selection in cancer and somatic tissues. Cell. 2018;173(7):1823 10.1016/j.cell.2018.06.001 29906452 PMC6005233

[47] Tomlinson IP, Novelli M, Bodmer W. The mutation rate and cancer. Proceedings of the National Academy of Sciences. 1996;93(25):14800–14803. 10.1073/pnas.93.25.14800 PMC26216 8962135

[48] Zack TI, Schumacher SE, Carter SL, Cherniack AD, Saksena G, Tabak B, et al Pan-cancer patterns of somatic copy number alteration. Nature genetics. 2013;45(10):1134 10.1038/ng.2760 24071852 PMC3966983

[49] Girirajan S, Campbell CD, Eichler EE. Human copy number variation and complex genetic disease. Annual review of genetics. 2011;45:203–226. 10.1146/annurev-genet-102209-163544 21854229 PMC6662611

[50] Krijgsman O, Carvalho B, Meijer GA, Steenbergen RD, Ylstra B. Focal chromosomal copy number aberrations in cancer—Needles in a genome haystack. Biochimica et Biophysica Acta (BBA)-Molecular Cell Research. 2014;1843(11):2698–2704. 25110350 10.1016/j.bbamcr.2014.08.001

[51] Cowell JK. Double minutes and homogeneously staining regions: gene amplification in mammalian cells. Annual review of genetics. 1982;16(1):21–59. 6760799 10.1146/annurev.ge.16.120182.000321

[52] Davis A, Gao R, Navin N. Tumor evolution: Linear, branching, neutral or punctuated? Biochimica et Biophysica Acta (BBA)-Reviews on Cancer. 2017;1867(2):151–161. 28110020 10.1016/j.bbcan.2017.01.003 PMC5558210

[53] Pham-Gia T, Turkkan N. Determination of the Beta distribution form its Lorenz curve. Mathematical and computer modelling. 1992;16(2):73–84.

[54] Wang Y, Waters J, Leung ML, Unruh A, Roh W, Shi X, et al Clonal evolution in breast cancer revealed by single nucleus genome sequencing. Nature. 2014;512(7513):155 10.1038/nature13600 25079324 PMC4158312

[55] Blum M, François O. Which random processes describe the tree of life? A large-scale study of phylogenetic tree imbalance. Systems biology. 2006;55(8):685–691. 10.1080/10635150600889625 16969944

[56] Aldous D. Probability distributions on cladograms In *Random discrete structure*. 1996; p. 1–18.

[57] Sainudiin R, Véber A. A Beta splitting model for evolutionary trees. Royal Society open science. 2016;3(160016). 10.1098/rsos.160016 PMC4892442 27293780

