# Supplementary figures and images for: Assessing the performance of methods for copy number aberration detection from single-cell DNA sequencing data
Source: PLoS Comput Biol. 2020 Jul 13;16(7):e1008012. doi: 10.1371/journal.pcbi.1008012 (PMC7377518; doi:10.1371/journal.pcbi.1008012)

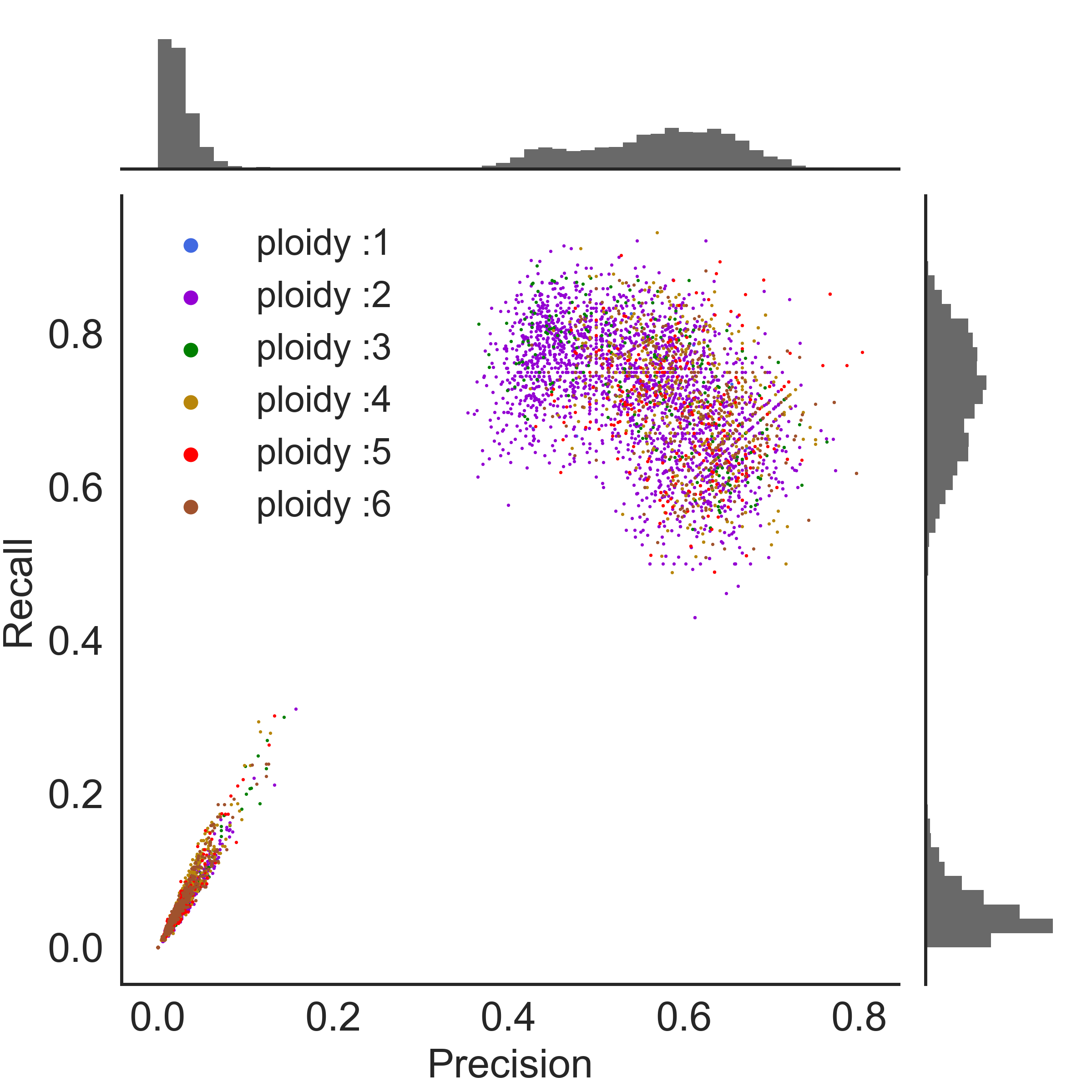

Supplement: S1 Fig — The color of the dots represent the ploidy that HMMcopy predicts. The upper and right histograms show the histogram of precision and recall, respectively, for all cells. (PNG) [file pcbi.1008012.s001.png]

(a) est. ploidy = 4

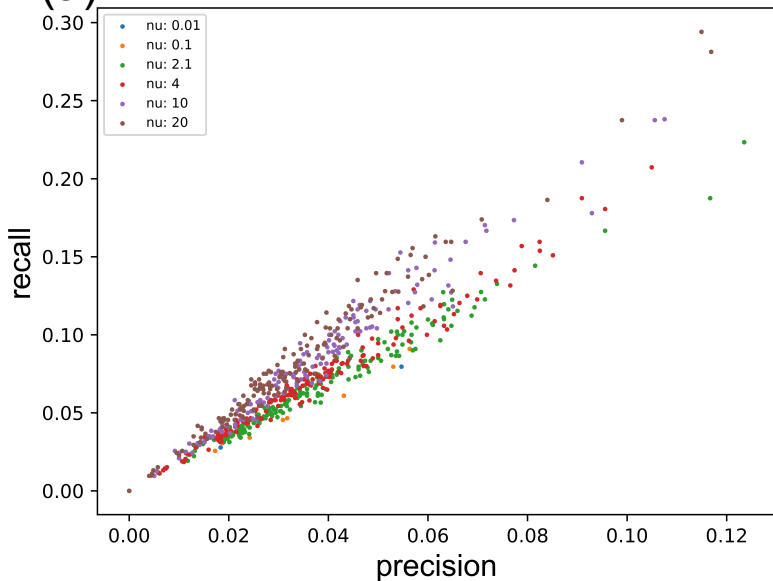

(b) est. ploidy = 5

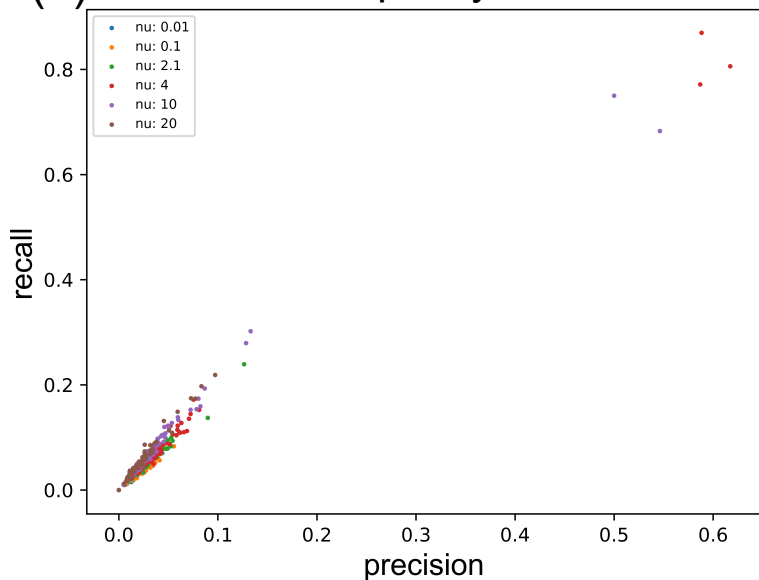

(c) est. ploidy = 6

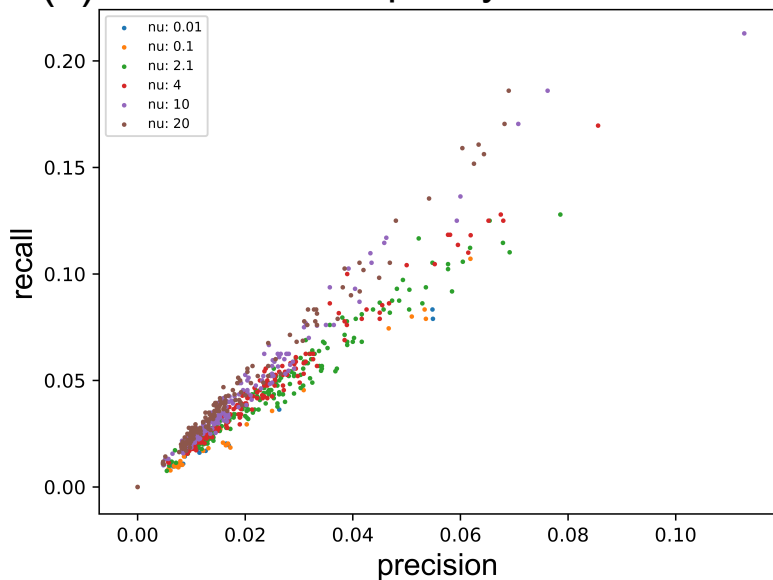

Supplement: S2 Fig — (a), (b) and (c) correspond to estimated ploidy 4, 5 and 6, respectively. (PDF) [file pcbi.1008012.s002.pdf]

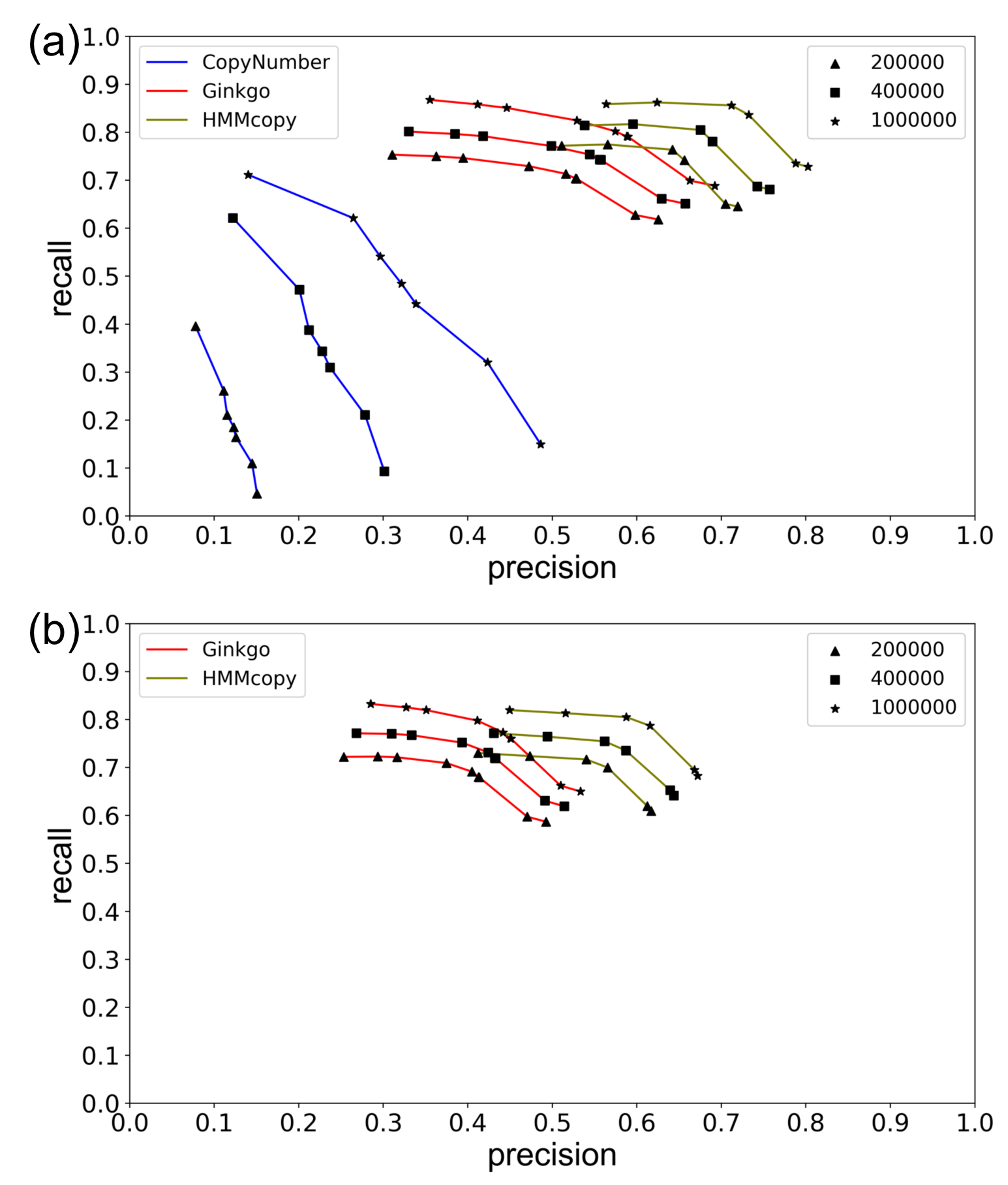

Supplement: S3 Fig — (a) Coarse-grained analysis results, and (b) fine-grained results for the second repetition. For each method, the results based on three thresholds of correctness are plotted. For HMMcopy, nu, which controls the suggested degree of freedom between states, was tuned to take on the values 0.01 (rightmost), 0.1, 2.1 (the tool’s default), 4, 10, and 20 (leftmost). For Ginkgo, alpha, which controls the significance level to accept a change point, was tuned to take on the values 1e-1000 (rightmost), 1e-100, 1e-10, 1e-5, 1e-4, 1e-3, 1e-2 (the tool’s default), 0.02 and 0.05 (leftmost). The dots corresponding to values 1e-5 and 1e-10 in coarse-grained analysis overlap. For CopyNumber, gamma, which is the weight of the penalty on changing a state, was tuned to take on the values 40 (rightmost, and the tool’s default), 10, 5, 4, 3, 2, and 1 (leftmost). (PNG) [file pcbi.1008012.s003.png]

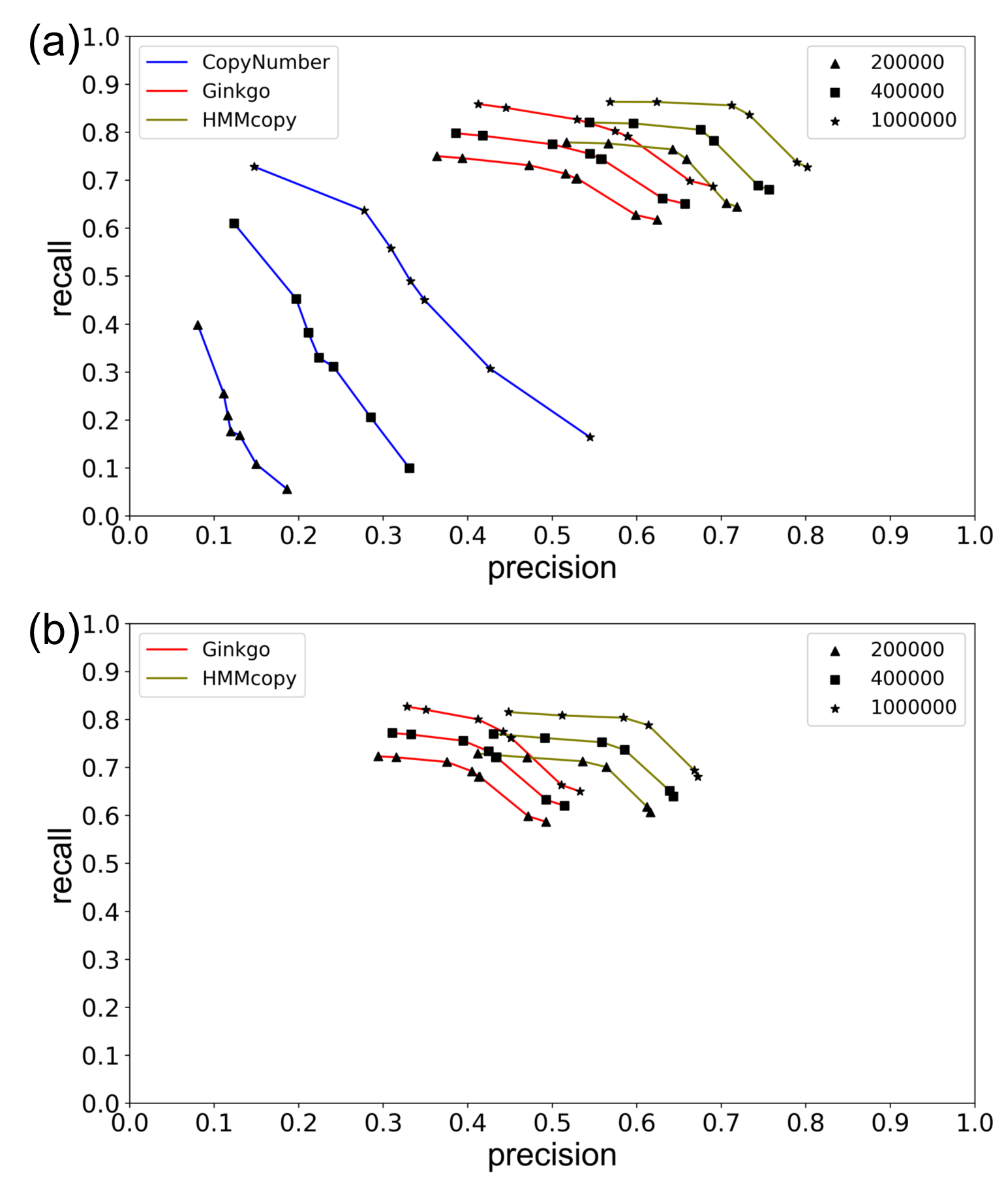

Supplement: S4 Fig — (a) Coarse-grained analysis results, and (b) fine-grained results for the third repetition. For each method, the results based on three thresholds of correctness are plotted. For HMMcopy, nu, which controls the suggested degree of freedom between states, was tuned to take on the values 0.01 (rightmost), 0.1, 2.1 (the tool’s default), 4, 10, and 20 (leftmost). For Ginkgo, alpha, which controls the significance level to accept a change point, was tuned to take on the values 1e-1000 (rightmost), 1e-100, 1e-10, 1e-5, 1e-4, 1e-3, 1e-2 (the tool’s default), 0.02 and 0.05 (leftmost). The dots corresponding to values 1e-5 and 1e-10 in coarse-grained analysis overlap. For CopyNumber, gamma, which is the weight of the penalty on changing a state, was tuned to take on the values 40 (rightmost, and the tool’s default), 10, 5, 4, 3, 2, and 1 (leftmost). (PNG) [file pcbi.1008012.s004.png]

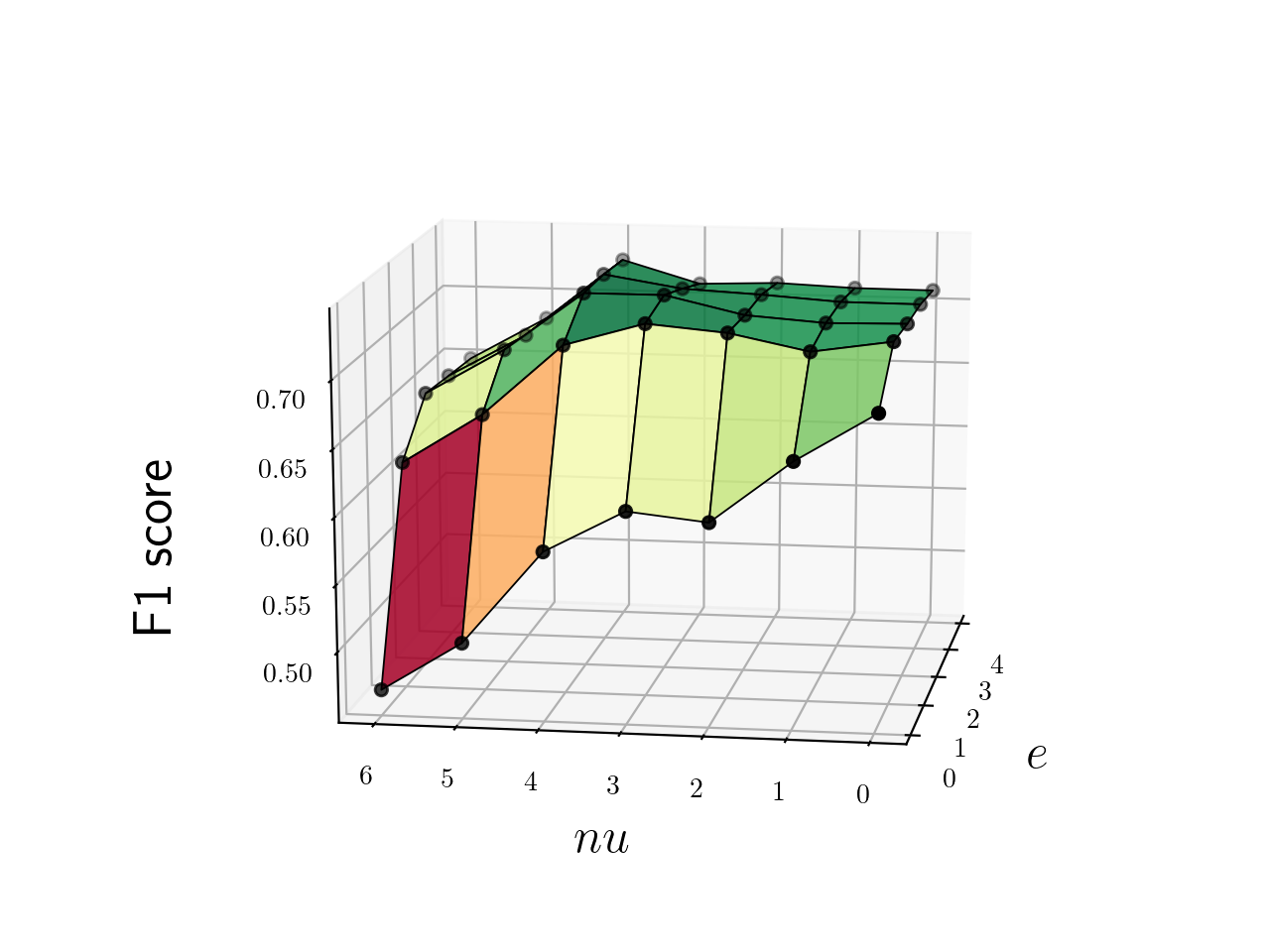

Supplement: S5 Fig — On nu, 0, 1, 2, 3, 4, 5 and 6 represent 0.01, 0.1, 1, 2.1 (default), 4, 10 and 20. On e, 0, 1, 2, 3, and 4 represent 0.99, 0.9999, 0.999999, 0.99999999 (default), and 0.9999999999. It can be seen when nu is 4, and e is ≥ 0.999999, F1 score reaches the maximum. (PNG) [file pcbi.1008012.s005.png]

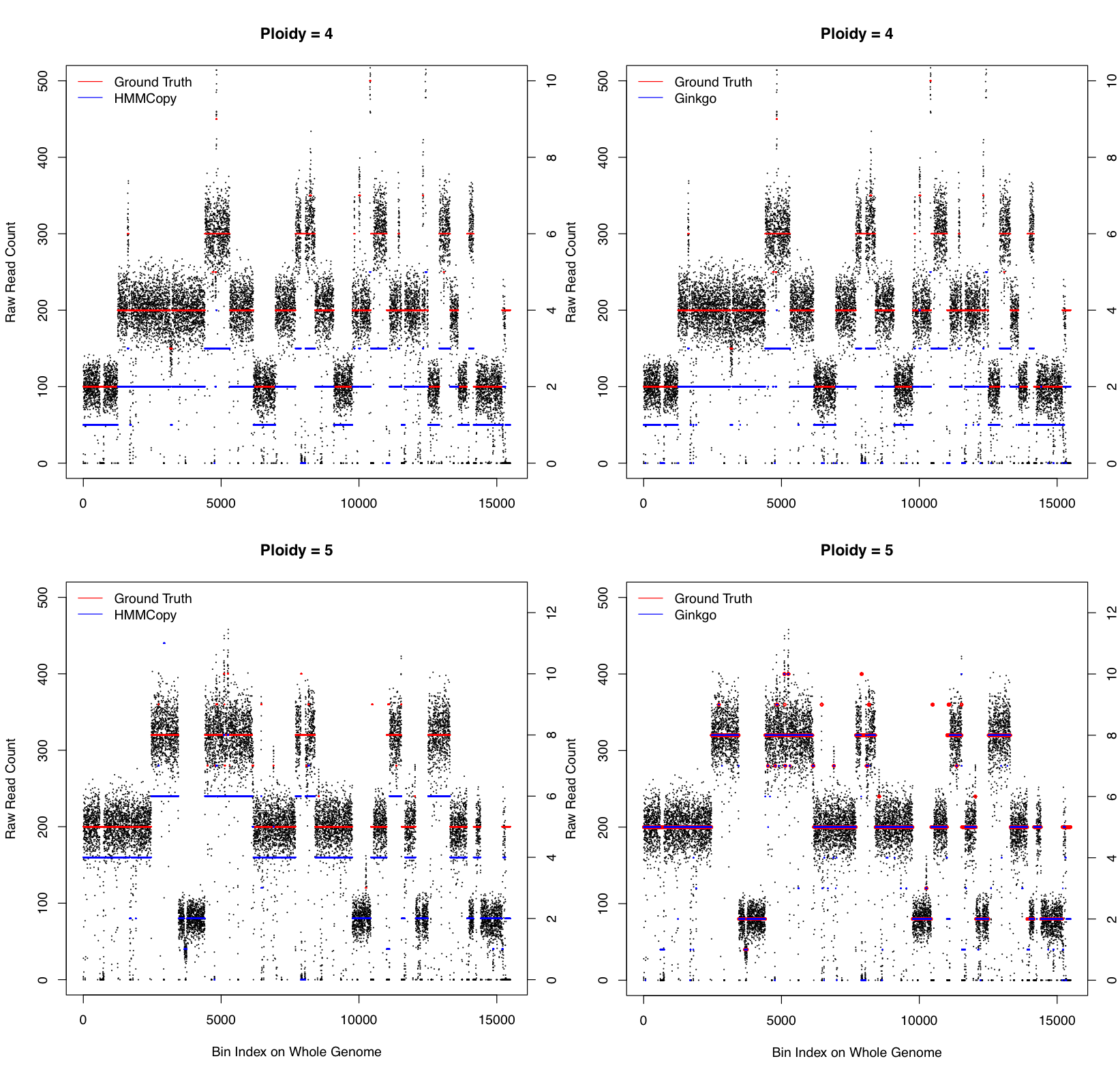

Supplement: S6 Fig — The left and right panels show HMMcopy and Ginkgo’s results, respectively. For each panel, X and primary Y axes represent the whole genome segmented into bins and the raw read count for each bin, respectively. The secondary Y axis represents the absolute copy number. Black dots represent the raw read count for each bin. Red and blue horizontal lines represent the ground truth of the absolute copy number and the inferred ones from HMMcopy and Ginkgo. Due to the lack of odd copy numbers, both HMMcopy and Ginkgo incorrectly predicted absolute copy number for the case of ploidy = 4. Due to the lack of intermediate copy number, HMMcopy incorreclty predicted absolute copy number for the case of ploidy = 5. Despite the lack of intermediate copy numbers, Ginkgo correctly predicted absolute copy number for the case of ploidy = 5 (red and blue lines overlap in this case). (PNG) [file pcbi.1008012.s006.png]

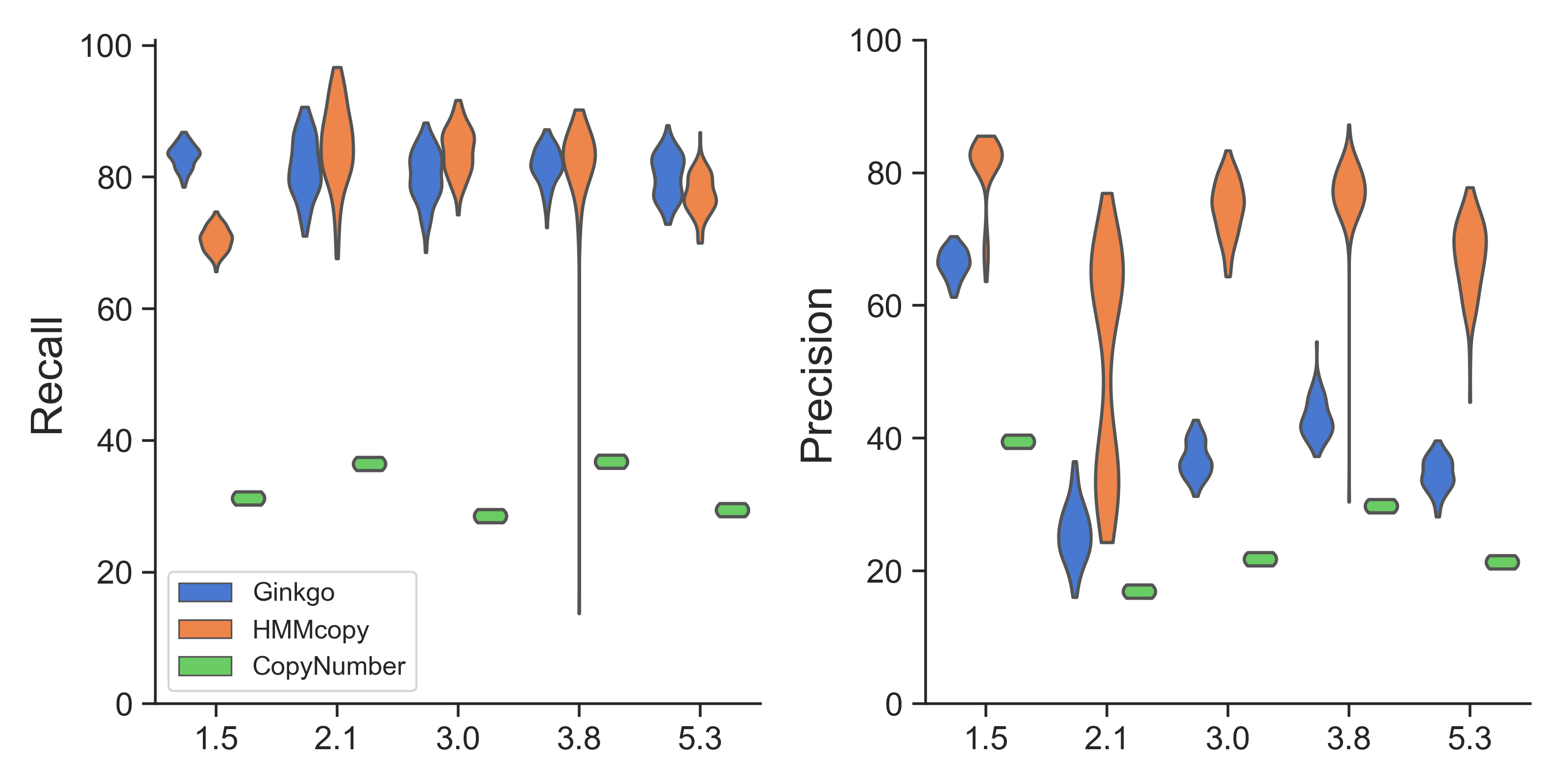

Supplement: S7 Fig — The ploidies of the simulated data were 1.5, 2.1, 3.0, 3.8, and 5.3. (PNG) [file pcbi.1008012.s007.png]

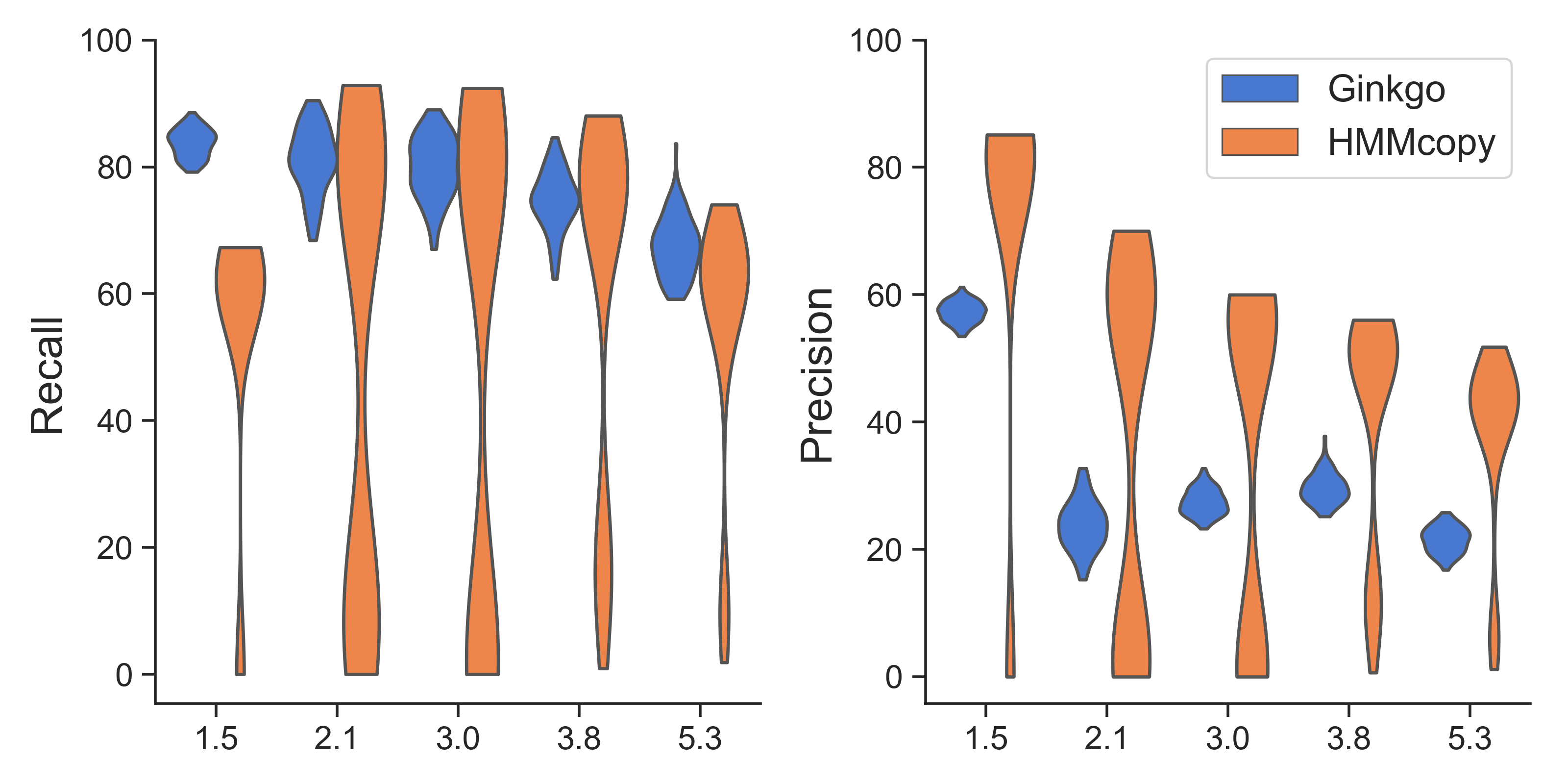

Supplement: S8 Fig — The ploidies of the simulated data were 1.5, 2.1, 3.0, 3.8, and 5.3. (PNG) [file pcbi.1008012.s008.png]

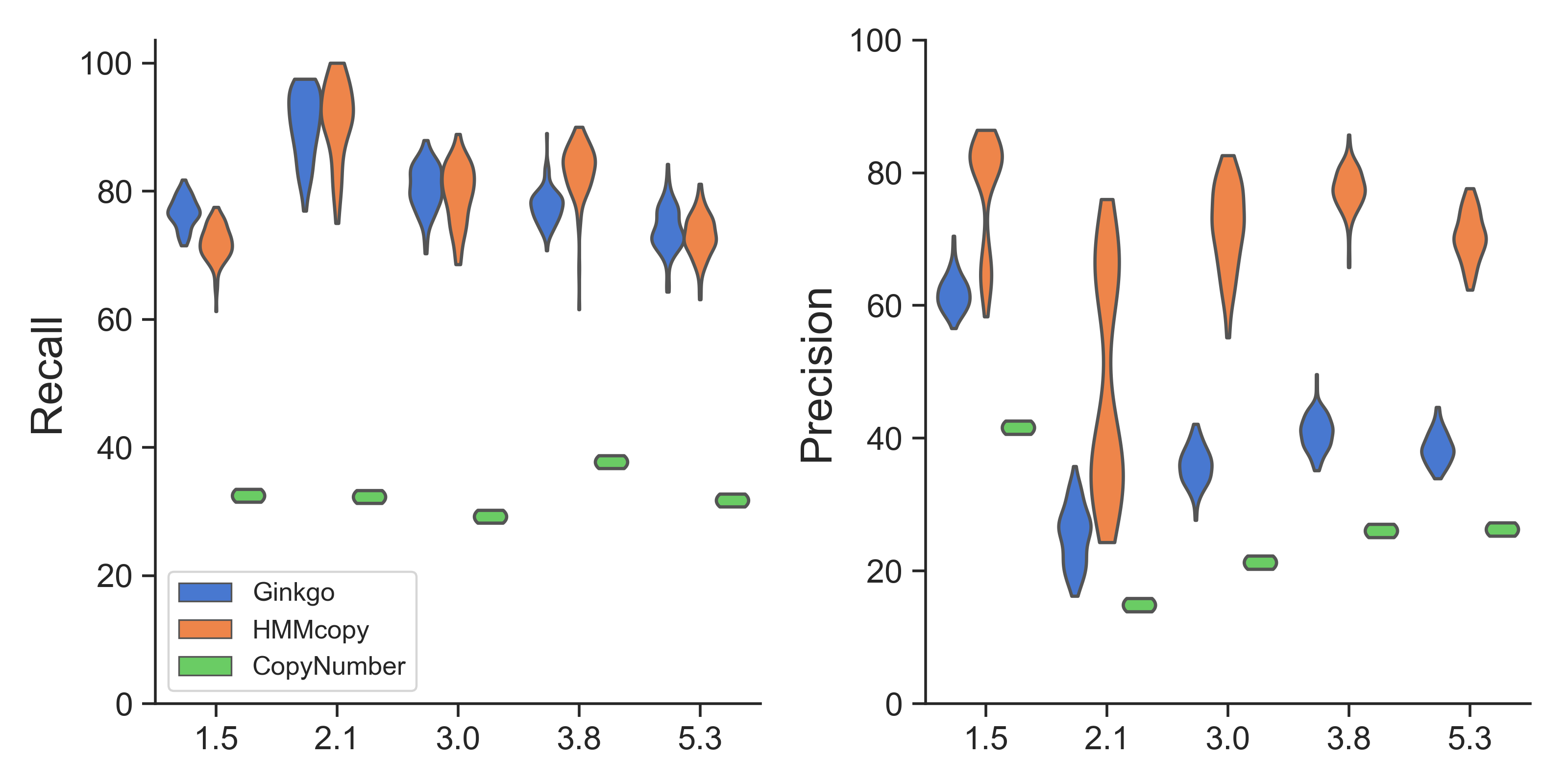

Supplement: S9 Fig — The ploidies of the simulated data were 1.5, 2.1, 3.0, 3.8, and 5.3. (PNG) [file pcbi.1008012.s009.png]

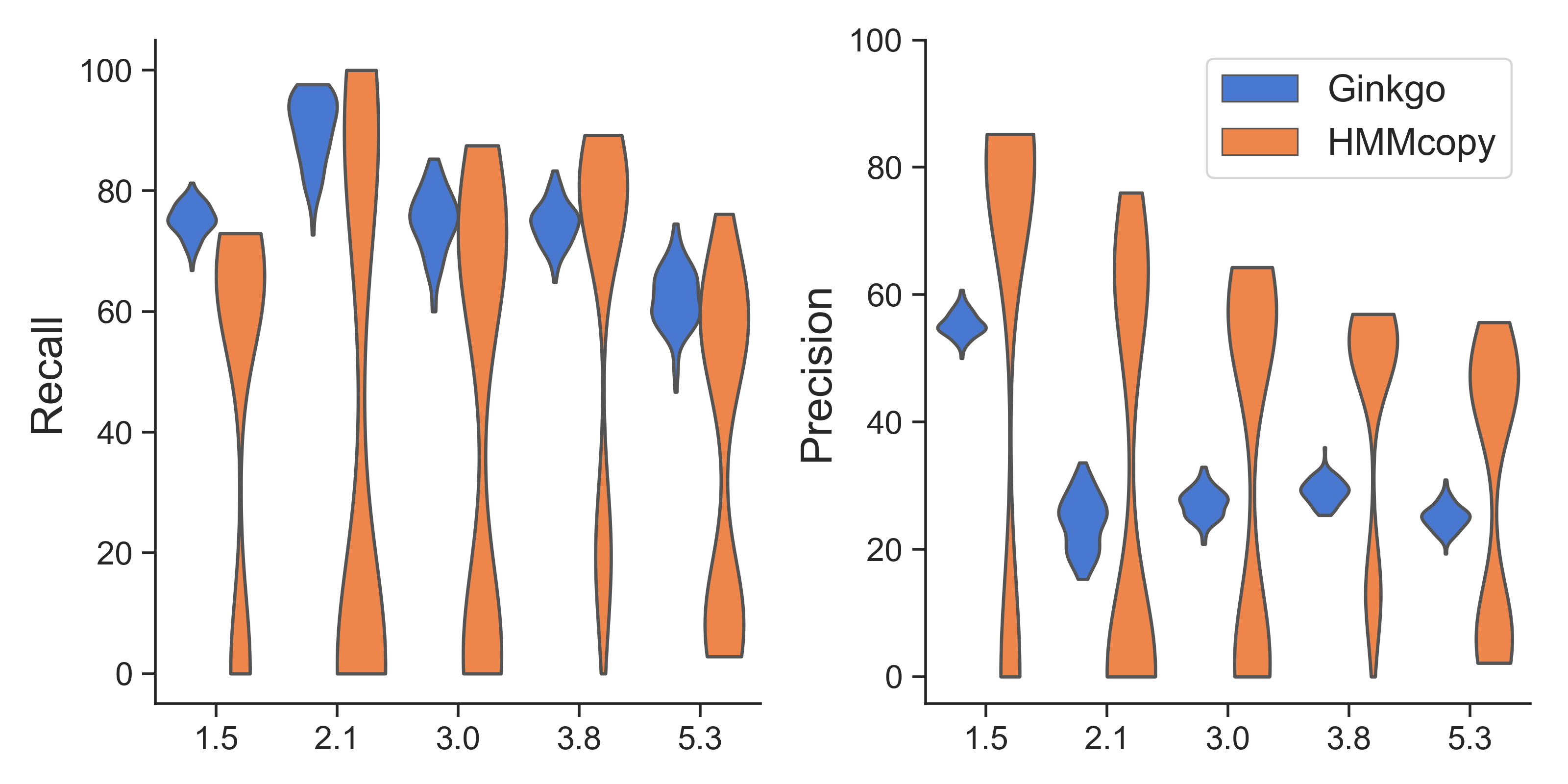

Supplement: S10 Fig — The ploidies of the simulated data were 1.5, 2.1, 3.0, 3.8, and 5.3. (PNG) [file pcbi.1008012.s010.png]

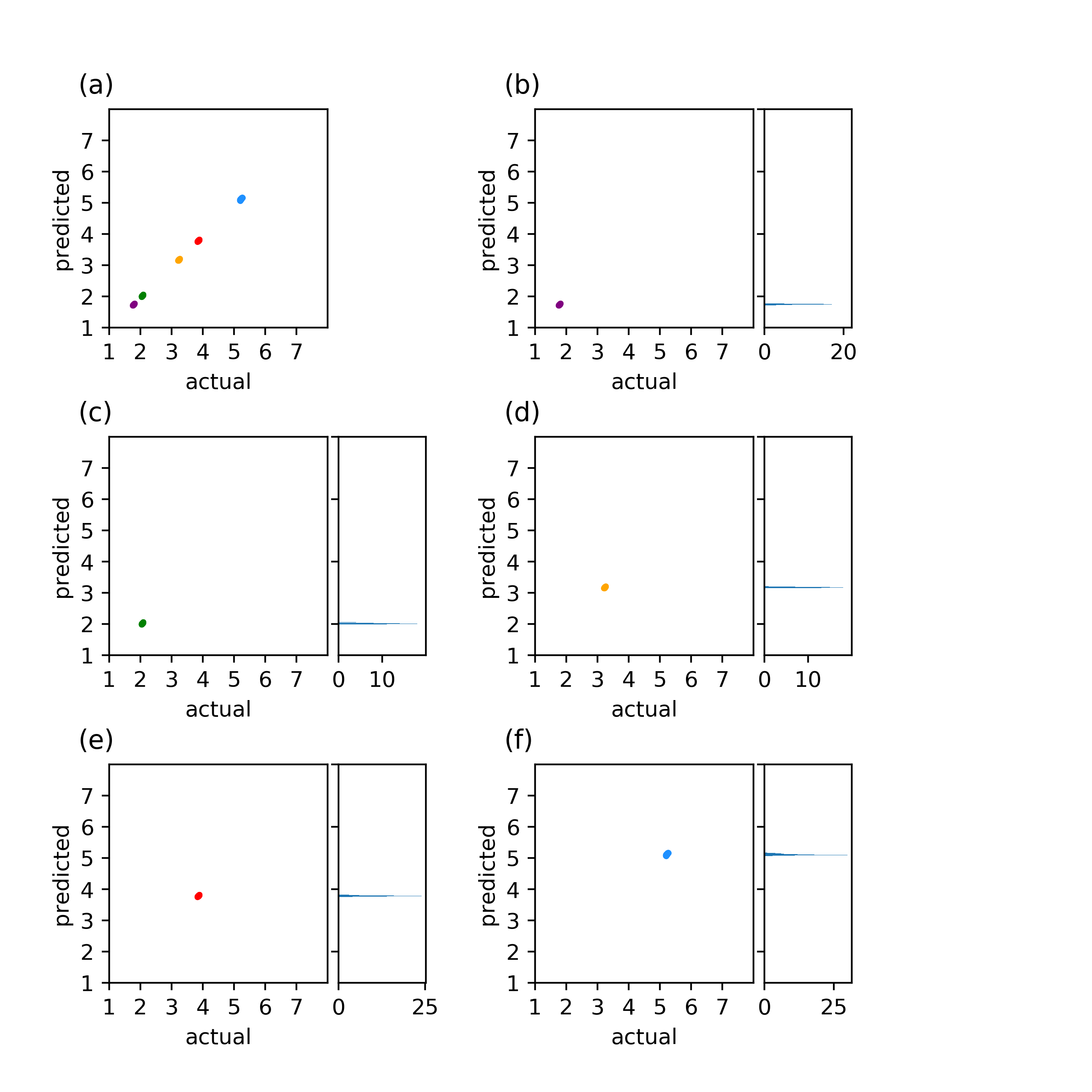

Supplement: S11 Fig — (a) A summary of all ploidies. Dots in purple, green, orange, red and blue represent the simulated ploidies at 1.5, 2.1, 3.0, 3.8 and 5.3. (b)-(f) Scatter plot of the predicted and actual ploidies for the five varying ploidies mentioned in (a) with their corresponding colors. Ploidy was calculated as the average copy number of the whole genome. On the right of each subplot, a histogram of the predicted ploidies is drawn to show the percentage of each predicted value. For (a)-(f), X and Y axis are the actual and predicted ploidies, respectively. (PNG) [file pcbi.1008012.s011.png]

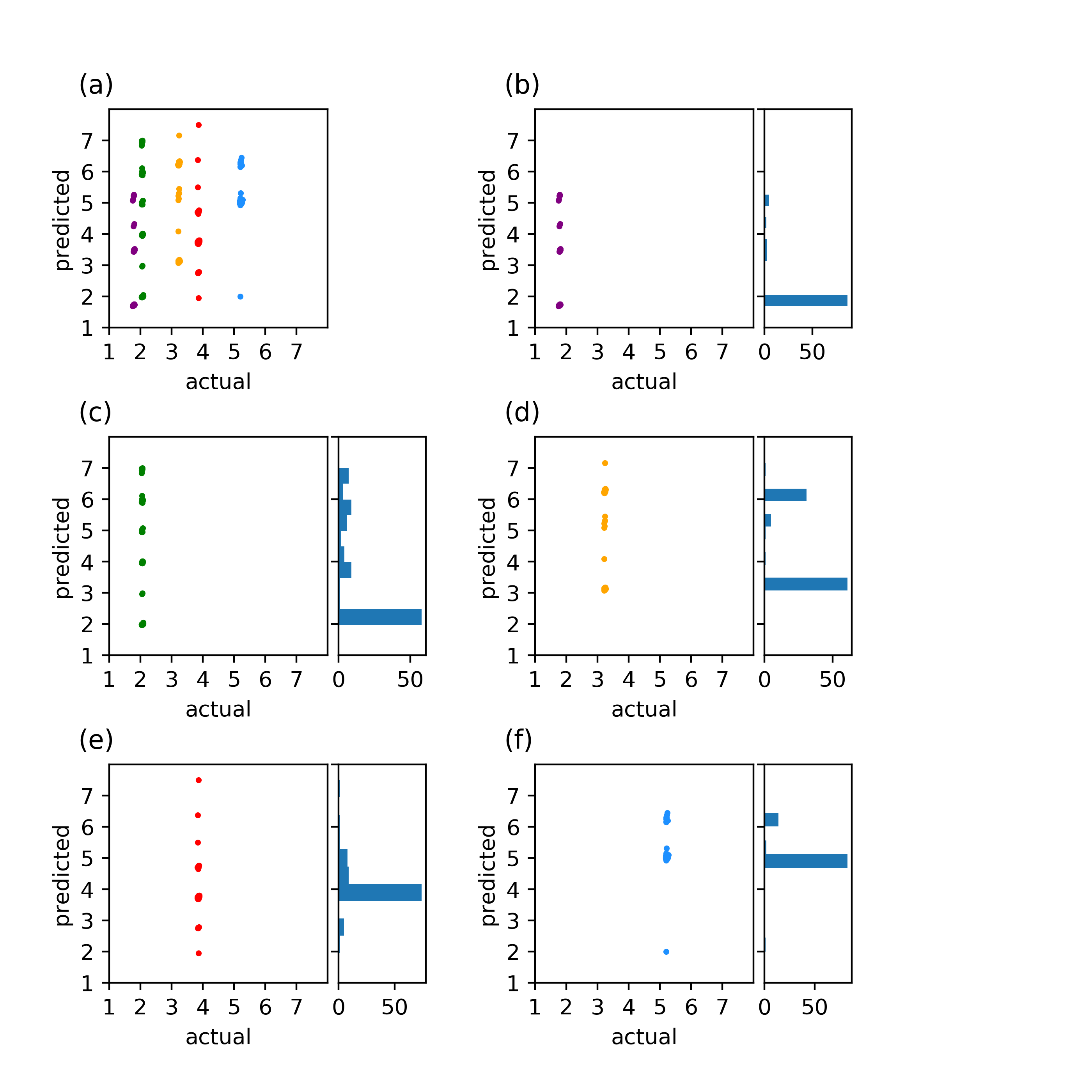

Supplement: S12 Fig — (a) A summary of all ploidies. Dots in purple, green, orange, red and blue represent the simulated ploidies at 1.5, 2.1, 3.0, 3.8 and 5.3. (b)-(f) Scatter plot of the predicted and actual ploidies for the five varying ploidies mentioned in (a) with their corresponding colors. Ploidy was calculated as the average copy number of the whole genome. On the right of each subplot, a histogram of the predicted ploidies is drawn to show the percentage of each predicted value. For (a)-(f), X and Y axis are the actual and predicted ploidies, respectively. (PNG) [file pcbi.1008012.s012.png]

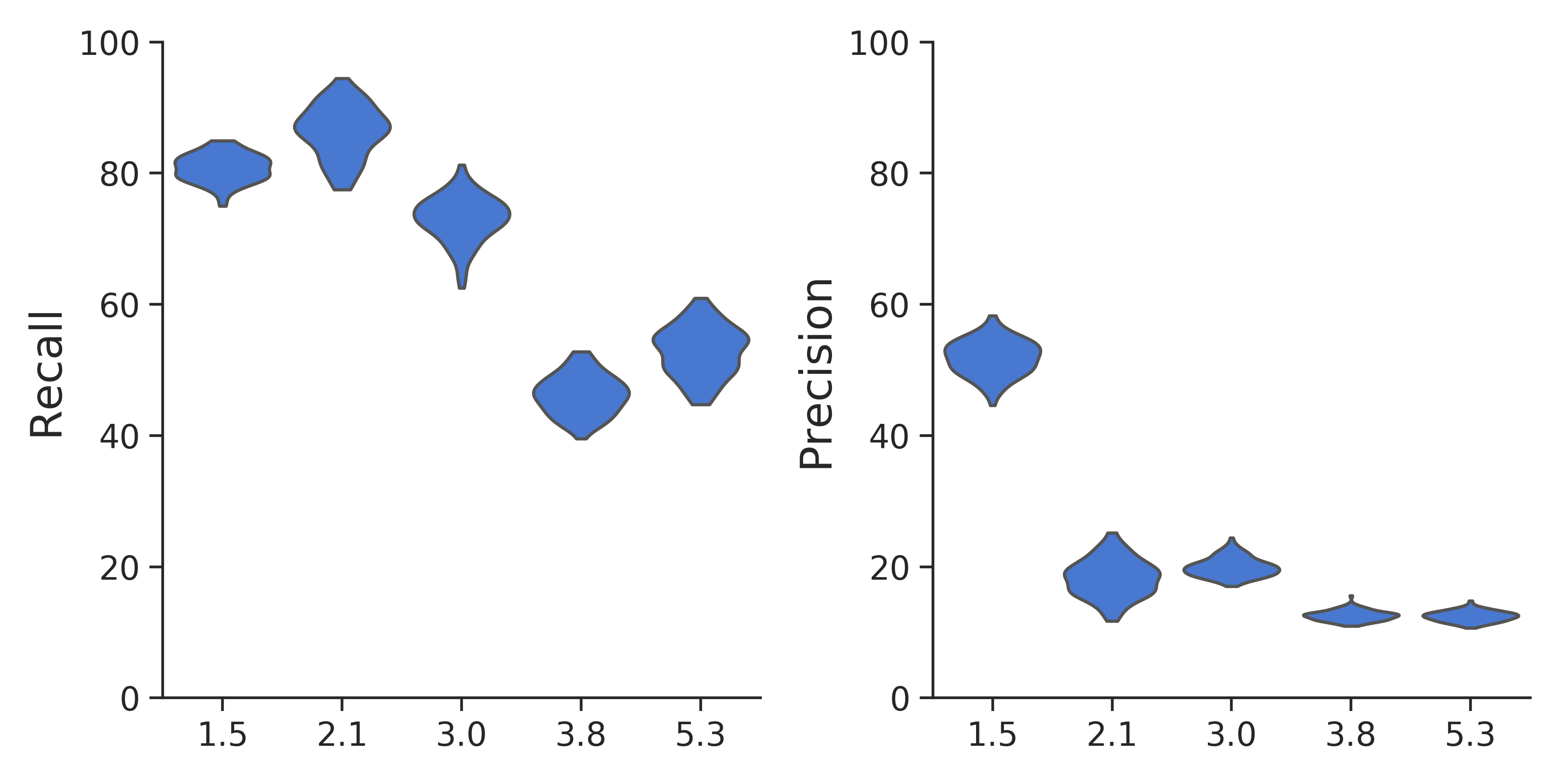

Supplement: S13 Fig — The ploidies of the simulated data were 1.5, 2.1, 3.0, 3.8, and 5.3. (PNG) [file pcbi.1008012.s013.png]

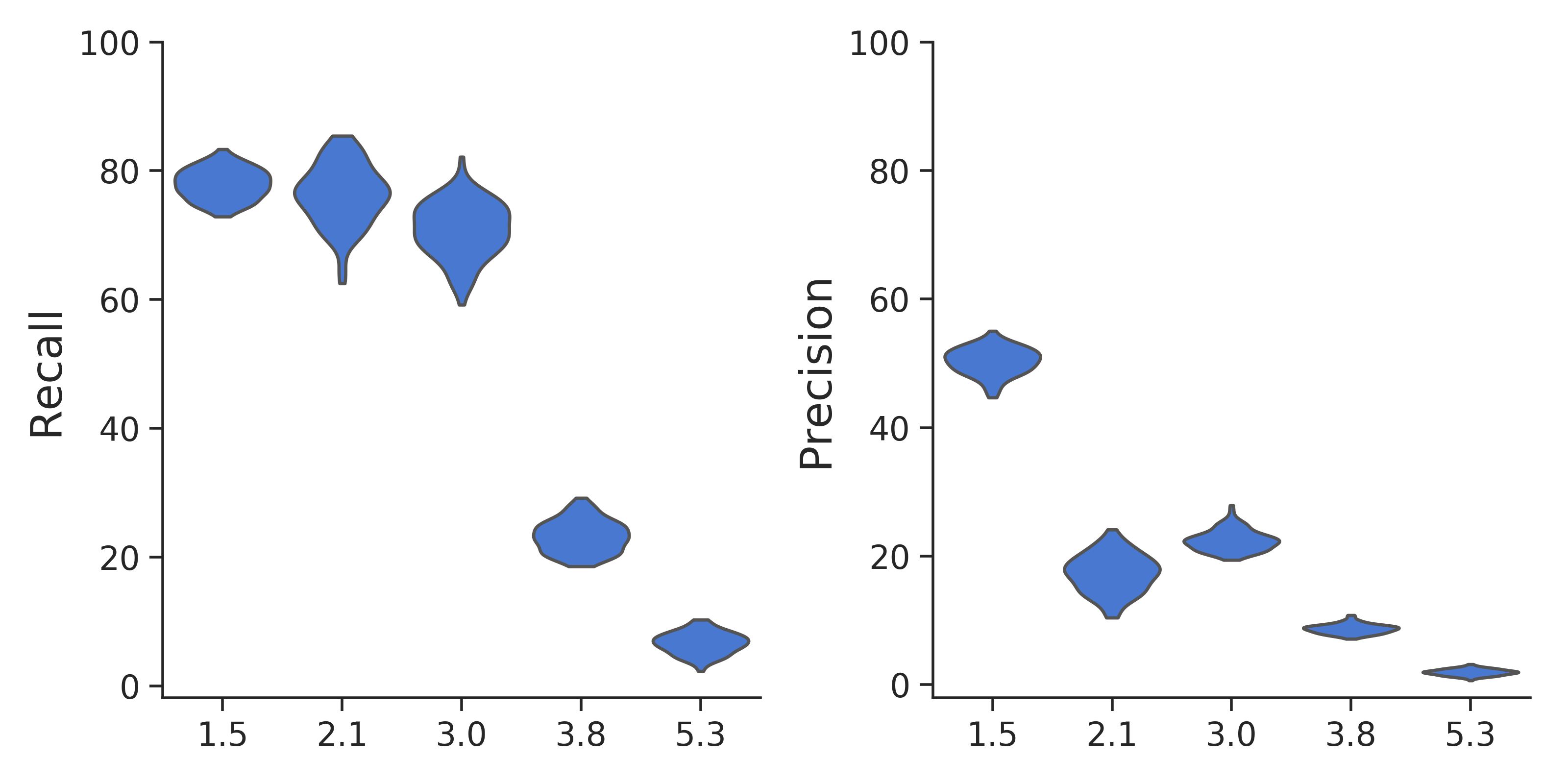

Supplement: S14 Fig — The ploidies of the simulated data were 1.5, 2.1, 3.0, 3.8, and 5.3. (PNG) [file pcbi.1008012.s014.png]

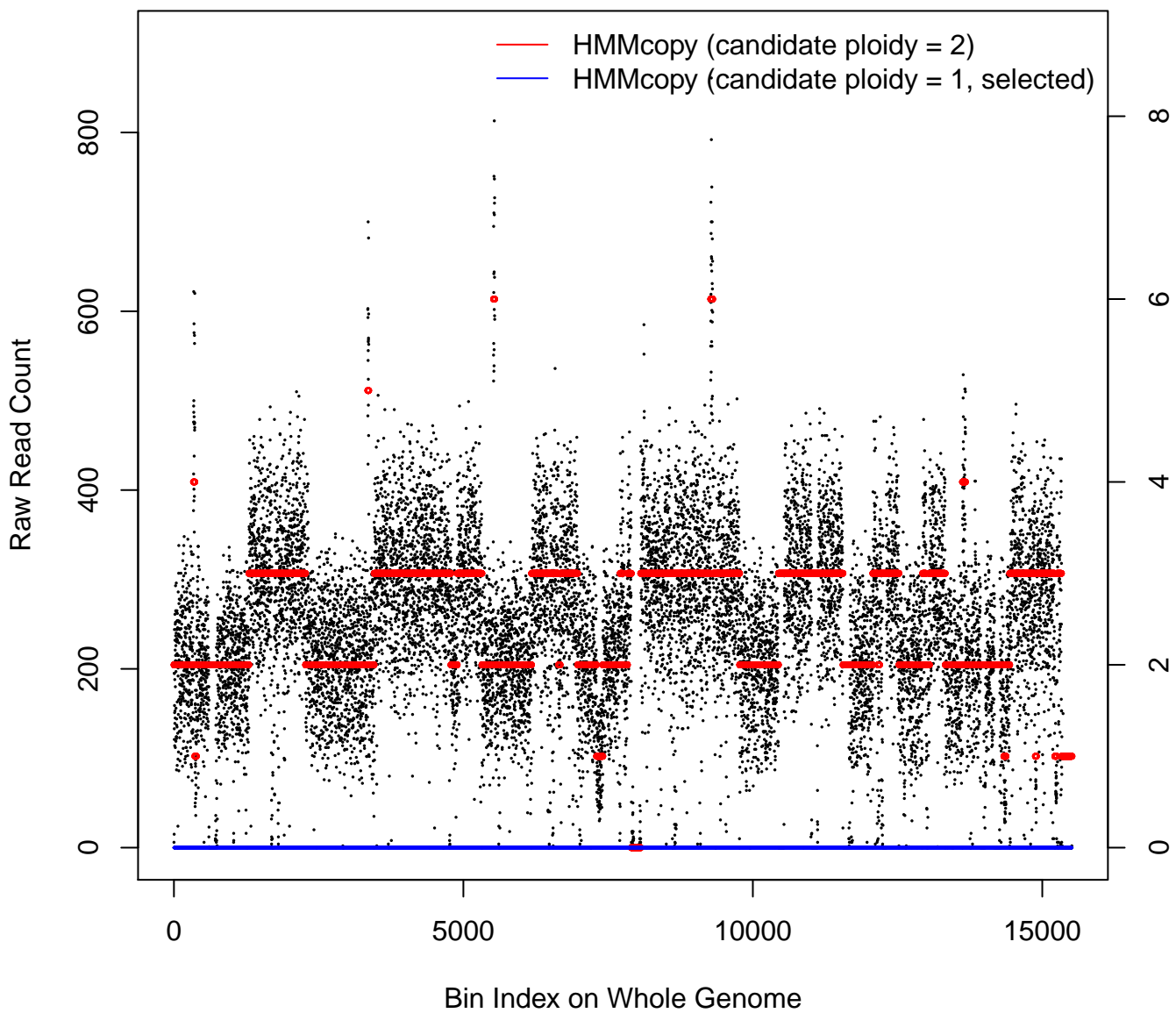

Supplement: S15 Fig — X and primary Y axes represent the whole genome segmented into bins and the raw read count for each bin, respectively. The secondary Y axis represents the absolute copy number. Black dots represent the raw read count for each bin. Red and blue horizontal lines represent the copy number profiles of HMMcopy on the condition when its ploidy estimation is 2 and 1, respectively, the latter of which was selected as the optimal ploidy by HMMcopy. (PDF) [file pcbi.1008012.s015.pdf]

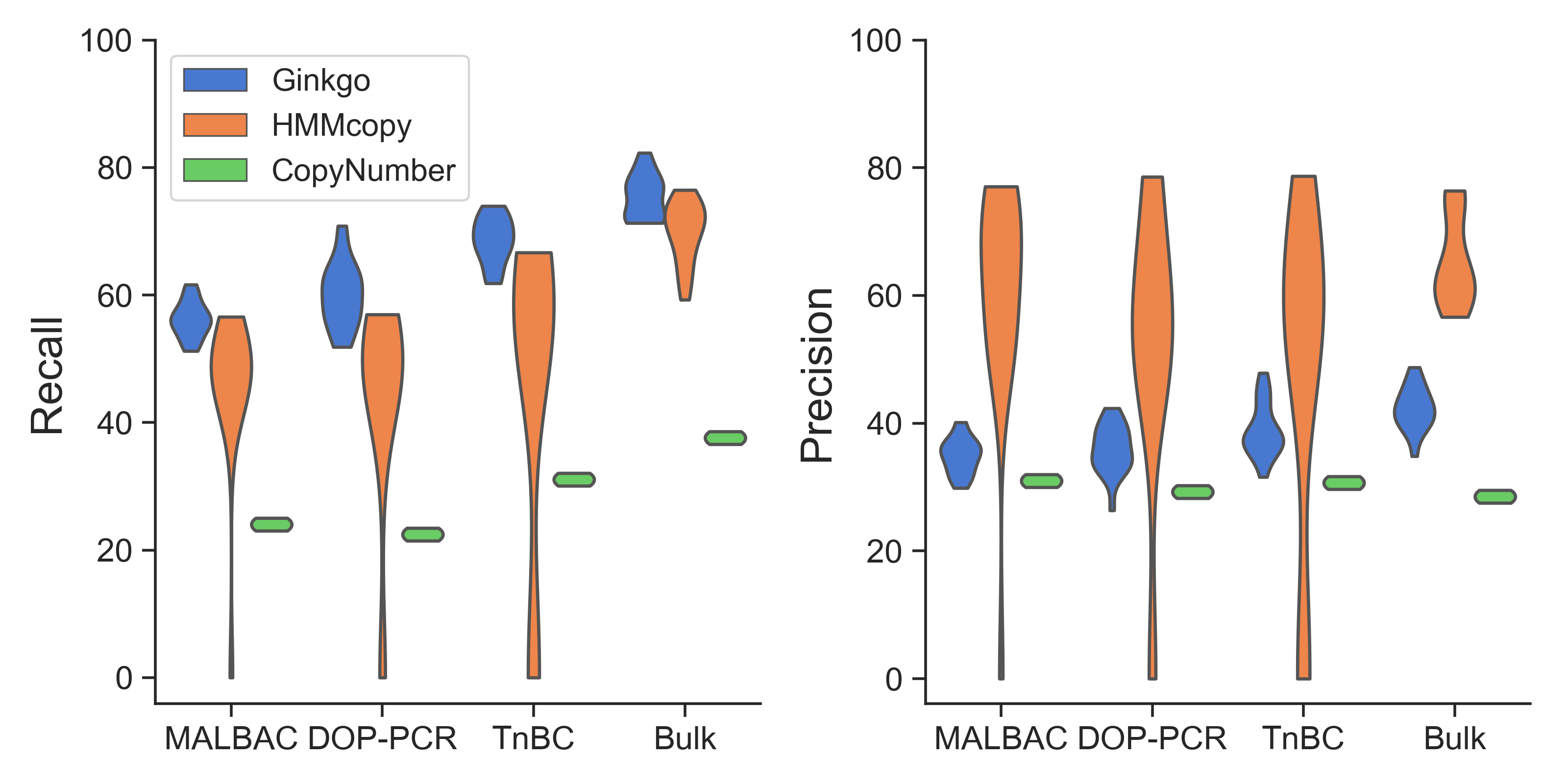

Supplement: S16 Fig — The coverages are varied to mimic those produced by MALBAC, DOP-PCR, TnBC and Bulk sequencing. (PNG) [file pcbi.1008012.s016.png]

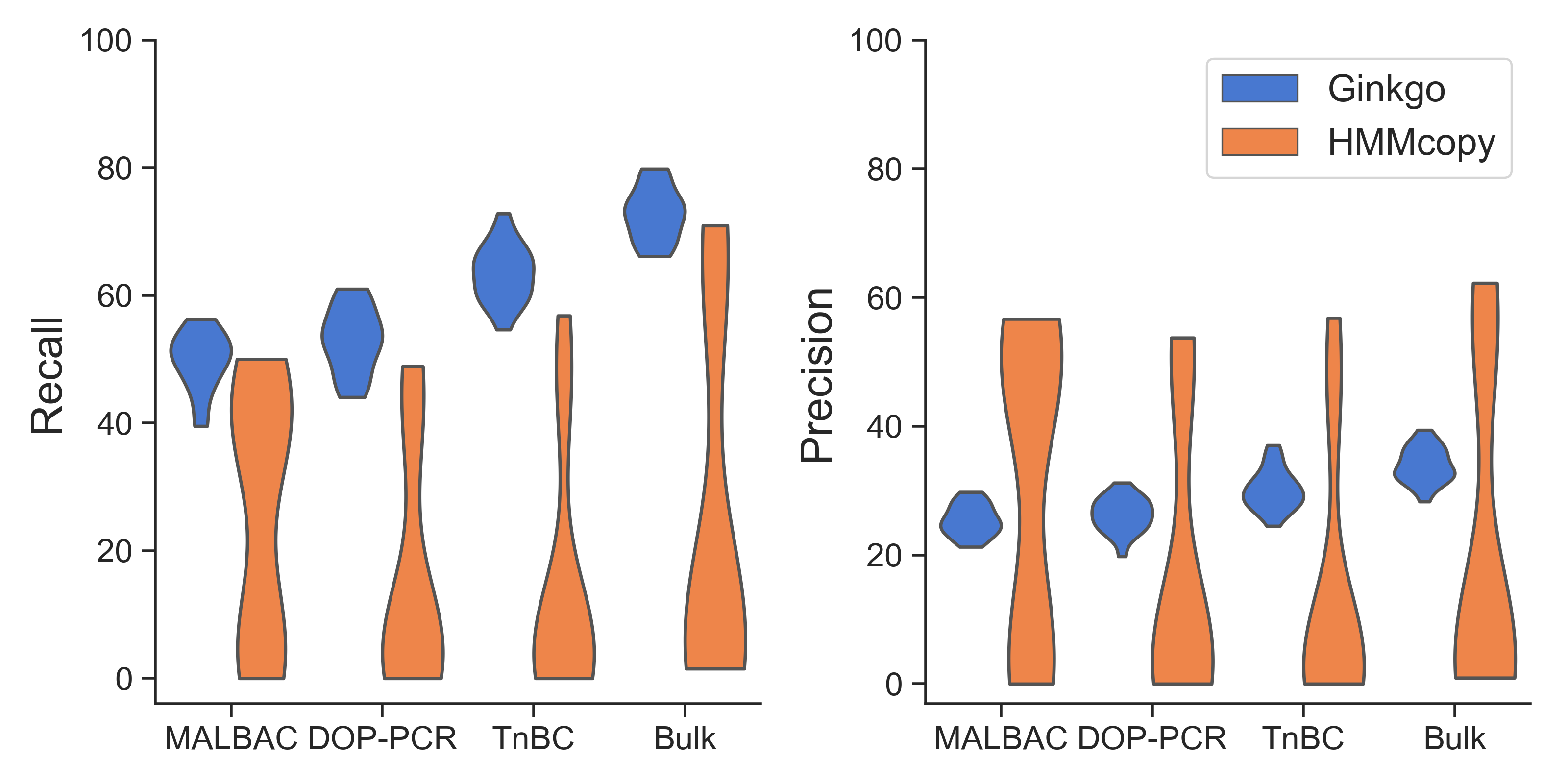

Supplement: S17 Fig — The coverages are varied to mimic those produced by MALBAC, DOP-PCR, TnBC and Bulk sequencing. (PNG) [file pcbi.1008012.s017.png]

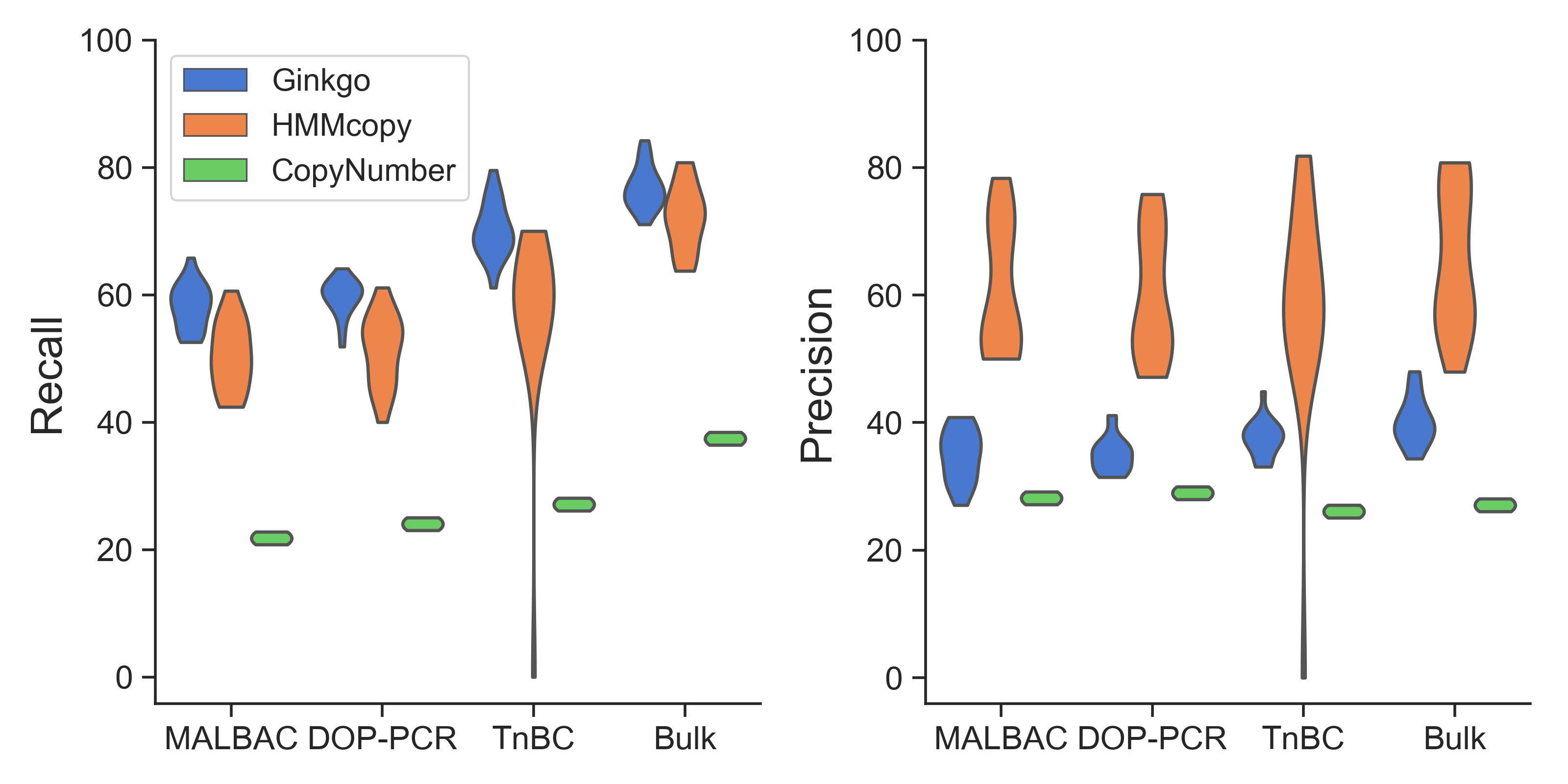

Supplement: S18 Fig — The coverages are varied to mimic those produced by MALBAC, DOP-PCR, TnBC and Bulk sequencing. (PNG) [file pcbi.1008012.s018.png]

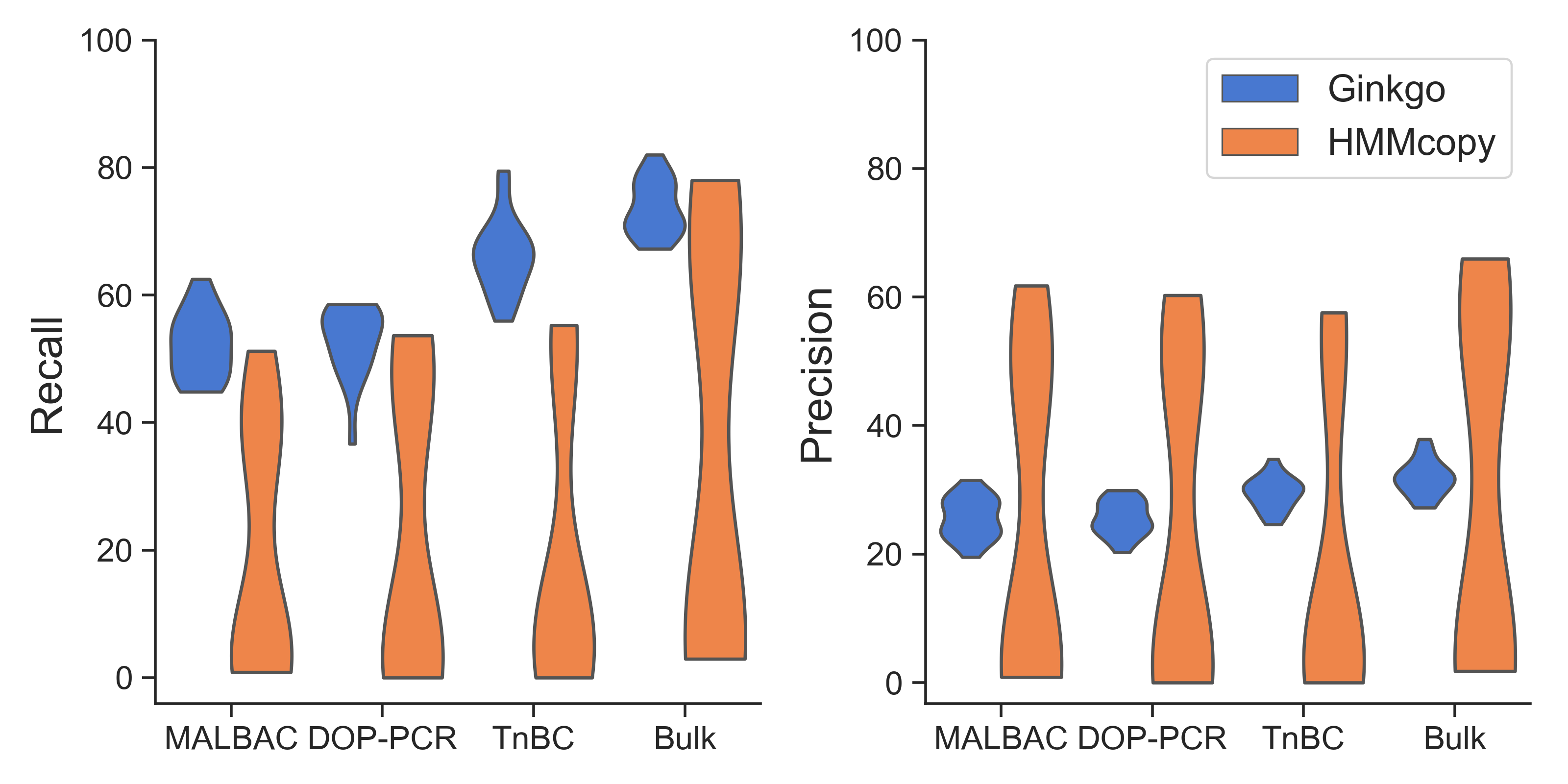

Supplement: S19 Fig — The coverages are varied to mimic those produced by MALBAC, DOP-PCR, TnBC and Bulk sequencing. (PNG) [file pcbi.1008012.s019.png]

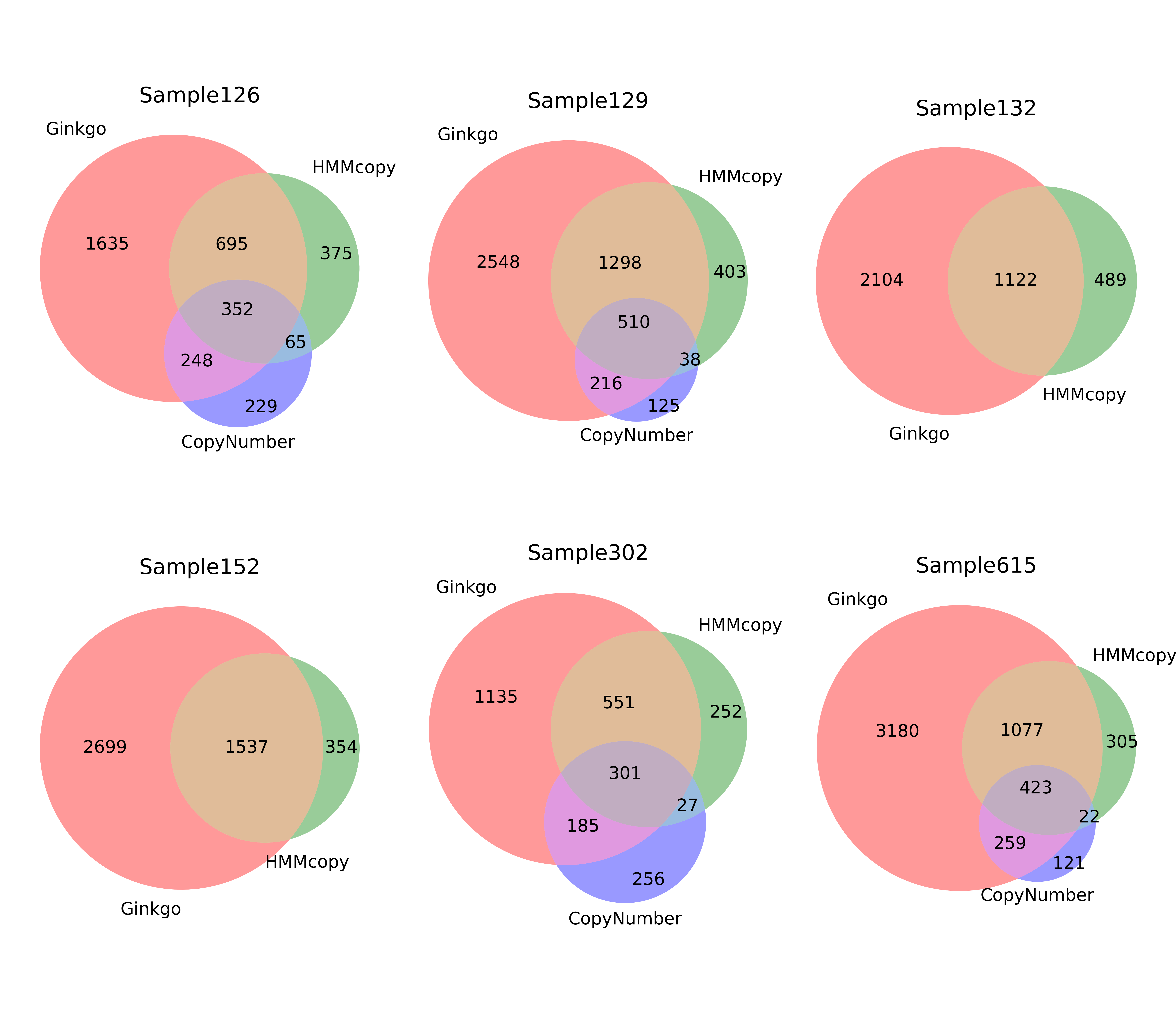

Supplement: S20 Fig — Breakpoints from two methods are counted as overlapped ones if they are within 400,000bp. (PNG) [file pcbi.1008012.s020.png]

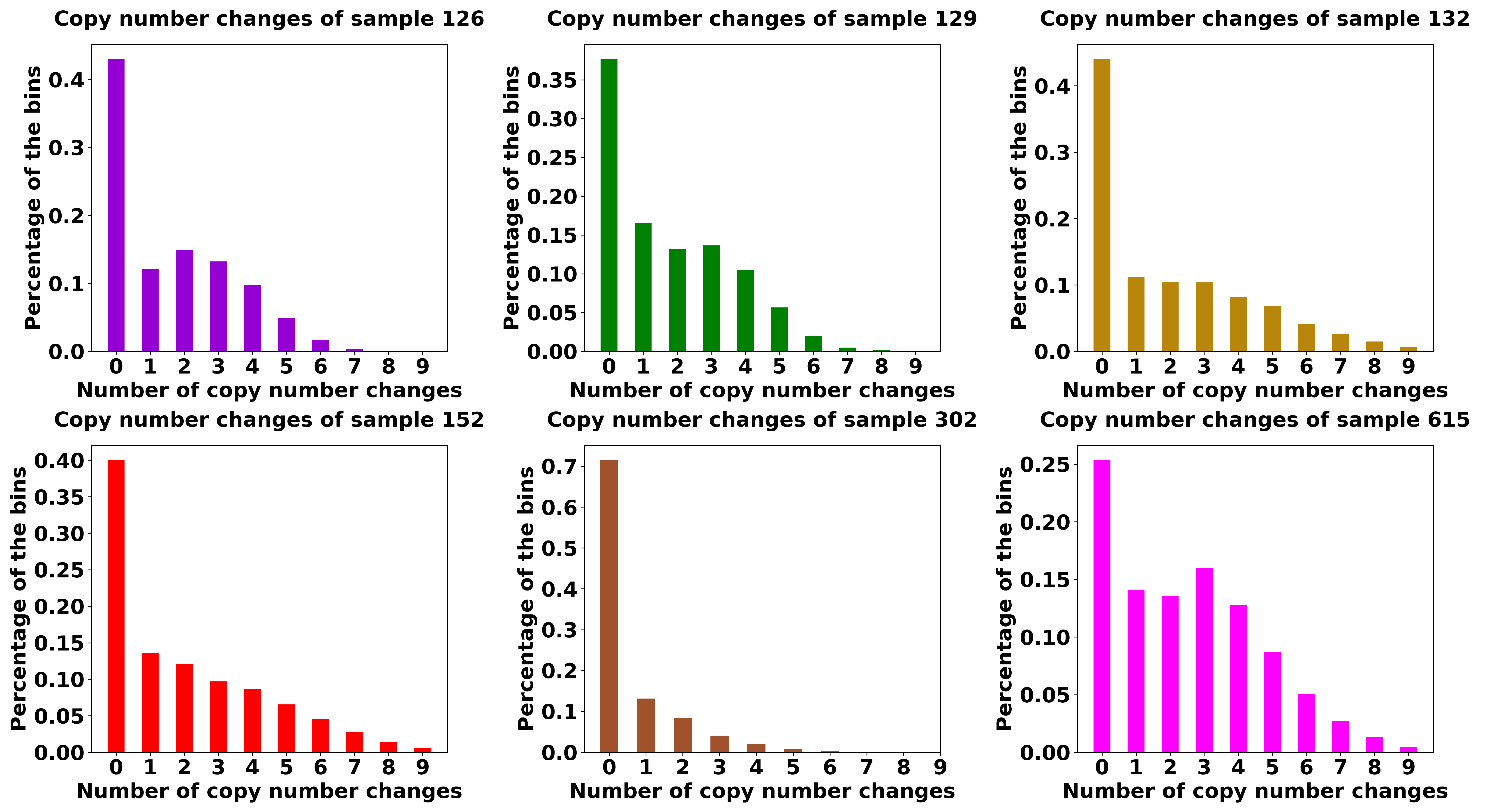

Supplement: S21 Fig — A maximum parsimony tree was inferred from the copy number profiles of the cells, and the minimum number of copy number changes per bin along all the branches of the tree was computed by parsimony analysis. The percentages of bins with each number of copy number changes are plotted. (PNG) [file pcbi.1008012.s021.png]

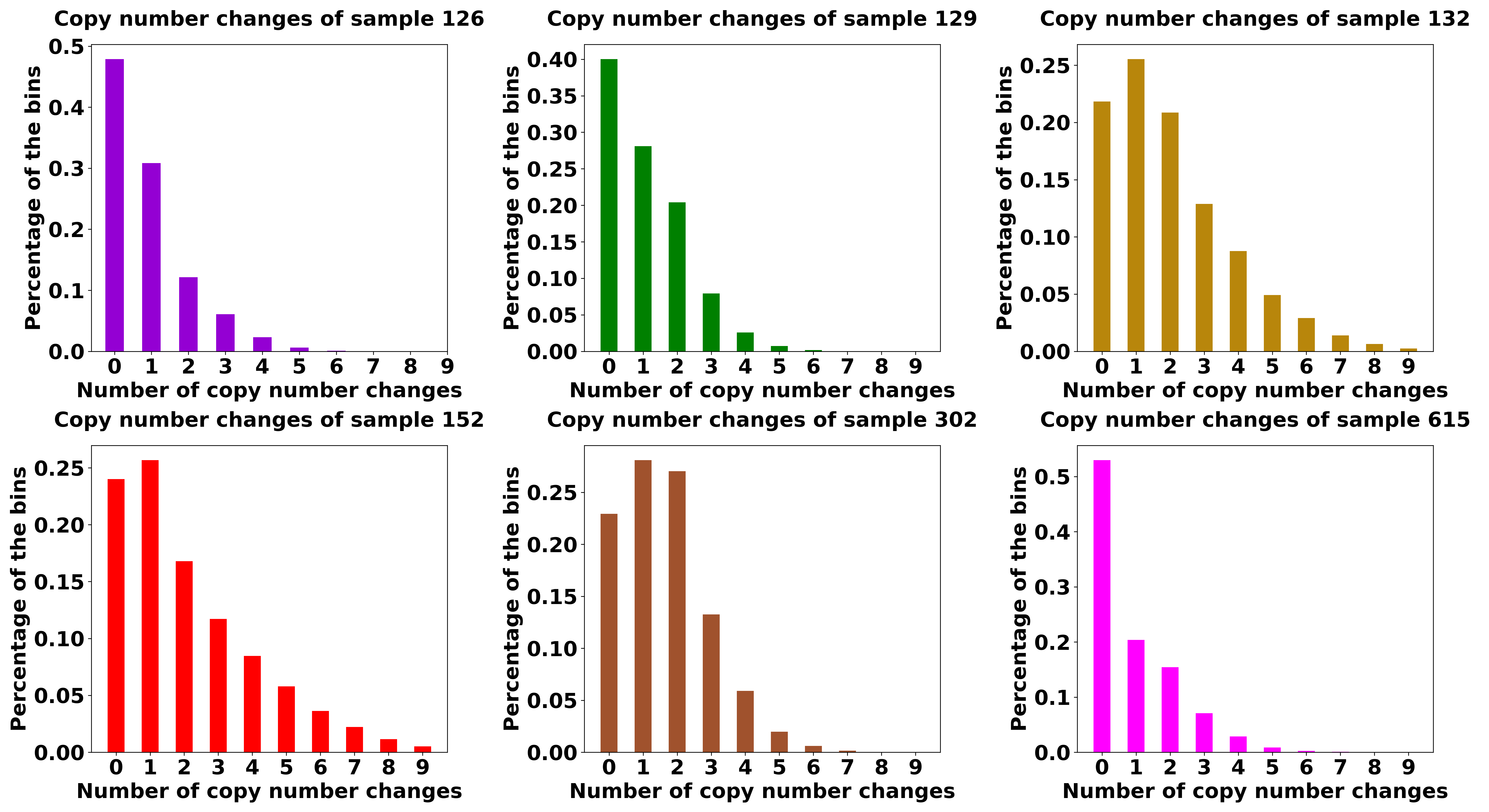

Supplement: S22 Fig — A maximum parsimony tree was inferred from the copy number profiles of the cells, and the minimum number of copy number changes per bin along all the branches of the tree was computed by parsimony analysis. The percentages of bins with each number of copy number changes are plotted. (PNG) [file pcbi.1008012.s022.png]

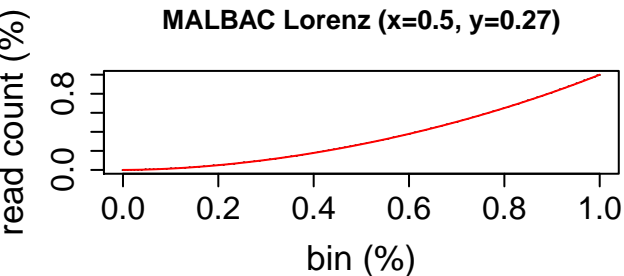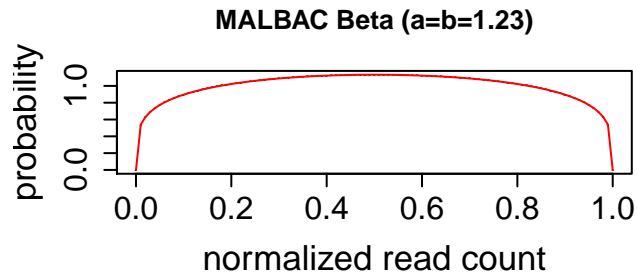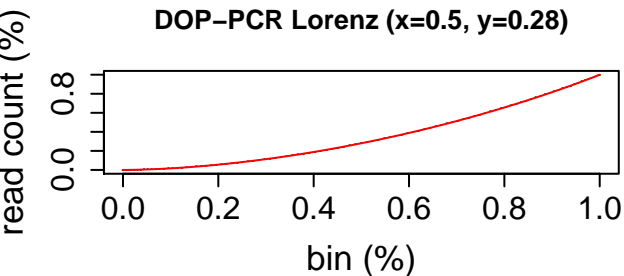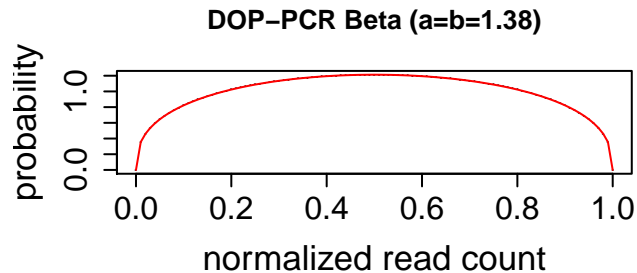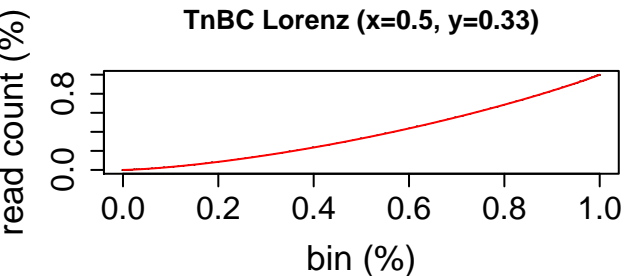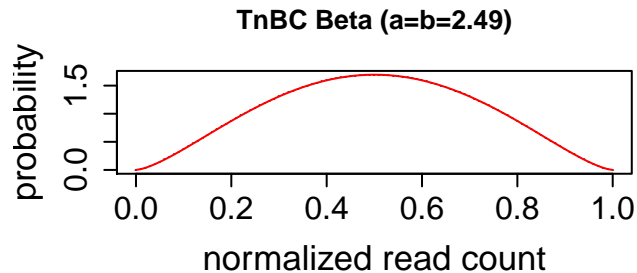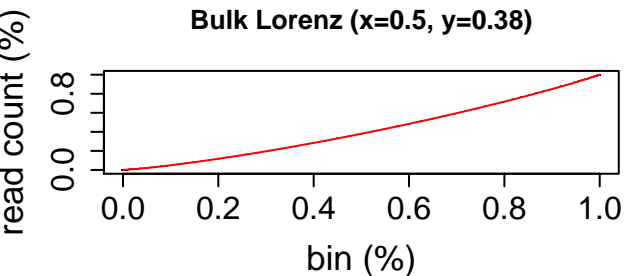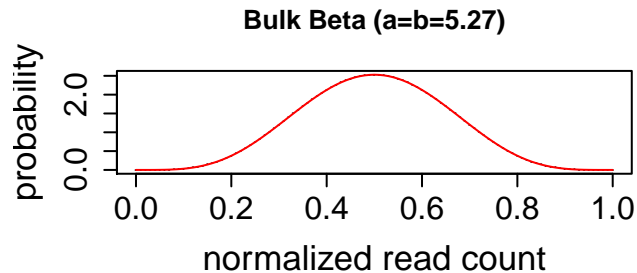

Supplement: S23 Fig — The four rows represent four sequencing technologies: MALBAC, DOP-BAC, TnBC and Bulk. The parameters of generating Lorenz curve were learned from [39]. The parameters of generating Beta distributions were learned from Eqs 1 and 2 in the main text. (PDF) [file pcbi.1008012.s023.pdf]
